# Divergent Fates of Hardjo Leptospires: Early Transcriptomic Response of *Leptospira interrogans* in an Ovine Dialysis Membrane Chamber Model

**DOI:** 10.1155/tbed/2998023

**Published:** 2026-04-09

**Authors:** Klaudia Dubniewicz, Laura Pardyak, Patrycja Walczak, Agnieszka Pietsch-Fulbiszewska, Artur Gurgul, Igor Jasielczuk, Tomasz Szmatoła, Zbigniew J. Arent

**Affiliations:** ^1^ Department of Infectious Diseases and Public Health, Faculty of Veterinary Medicine, University of Agriculture, Krakow, 30–248, Poland, uaf.edu.pk; ^2^ Department of Diagnostics and Clinical Sciences, Faculty of Veterinary Medicine, University of Agriculture, Krakow, 30–248, Poland, uaf.edu.pk; ^3^ Department of Basic Sciences, Faculty of Veterinary Medicine, University of Agriculture, Krakow, 30–248, Poland, uaf.edu.pk

**Keywords:** dialysis membrane chamber model, Hardjo, *Leptospira interrogans*, ovine, transcriptome

## Abstract

*Leptospira interrogans* serovar Hardjo and *Leptospira borgpetersenii* serovar Hardjo are major bovine‐ and ovine‐adapted pathogens that differ markedly in genome content. To investigate early events during host exposure, we compared the survival of both species using an ovine peritoneal dialysis membrane chamber (DMC) model, which exposes leptospires to simultaneous nutrient restriction, temperature and osmotic shifts and small host‐derived immune factors such as antimicrobial peptides (AMPs). *L. borgpetersenii* failed to survive 24 h of in vivo exposure, whereas *L. interrogans* persisted and remained viable. RNA sequencing of two *L. interrogans* strains revealed extensive transcriptional reprogramming, with 1066 differentially expressed genes (DEGs). Early host exposure triggered coordinated remodelling of membrane‐associated proteins, including strong induction of adhesins (LigA/B and LenC/F) and other lipoproteins, accompanied by metabolic reprogramming that redirects cellular resources towards conservation and membrane reorganisation. Iron‐related genes were also differentially regulated, and *L. borgpetersenii* showed reduced survival under in vitro iron‐limiting conditions. A substantial proportion of downregulated genes belonged to pathways governing signal transduction, chemotaxis and motility, indicating early suppression of selected sensory and locomotive functions. Many of the genes most strongly regulated under experimental stress conditions lack orthologues in the *L. borgpetersenii* genome, suggesting that genome reduction has eliminated factors required for survival of the stressors encountered in this model, which further implies that this species relies on distinct early‐phase survival strategies that do not support extracellular persistence under the conditions tested. Our findings show that *L. interrogans* serovar Hardjo rapidly modulates cellular networks to promote early survival in body fluids or within the extracellular matrix.

## 1. Introduction

*Leptospira* is a genus of spirochaete bacteria that exhibits substantial serological and genetic diversity and is classified into saprophytic and pathogenic species. Comparative genomic studies have identified saprophytic lineages (S1 and S2) and pathogenic groups: P1^+^ (high virulence), P1^−^ (low virulence) and P2 (intermediate pathogenicity) [1, 2]. Pathogenic *Leptospira* are believed to have evolved from saprophytic ancestors, losing genes necessary for environmental survival while acquiring mechanisms that support survival within hosts during infection [3].

Most pathogenic *Leptospira* strains isolated from mammals belong to the P1^+^ group. These strains typically cause chronic, asymptomatic infections in reservoir hosts, while in incidental hosts they often lead to acute disease that can progress to multi‐organ failure without prolonged colonisation [4, 5]. Chronic infections in farm animals are a major concern because, even in the absence of obvious clinical signs, they can have substantial economic impact. Cattle and sheep are well‐established reservoir hosts of *Leptospira* serovar (sv.) Hardjo, which can be classified into two species: *L. interrogans* and *L. borgpetersenii*, previously referred to as Hardjo Prajitno and Hardjo Bovis, respectively [6–10]. Both have the ability to colonise and persist not only in the renal tubules but also in the reproductive tract, leading to infertility, abortion, stillbirth, weak offspring and retained foetal membranes. While both species show similar tissue tropism, clinical observations suggest differences in disease severity. *L. interrogans* sv. Hardjo infections tend to be more acute and more frequently affect incidental hosts [11]. Although this species was predominant in Europe in the 1970 and 1980s, more recent data indicate a shift in prevalence towards *L. borgpetersenii* sv. Hardjo, likely due to its greater adaptation to cattle and sheep [6].

Whole‐genome comparisons have shown that *L. interrogans* has retained genes that facilitate environmental survival, enabling indirect transmission. In contrast, *L. borgpetersenii* has undergone extensive genome reduction, losing many such genes and relying more on direct transmission between hosts [12–15]. These genomic differences are likely to influence not only transmission dynamics but also potentially affect host–pathogen interactions. Subsequent genomic studies further characterised the gene content and structural organisation of *L. interrogans* and *L. borgpetersenii*, providing deeper insight into lineage‐specific gene repertoires and patterns of genome reduction [16–19]. While these studies defined the genomic framework underlying divergence between the two Hardjo lineages, the functional consequences of these differences during early host exposure remain incompletely understood.

Pathogenic leptospires possess unique features, including atypical lipopolysaccharides (LPS), robust oxidative stress defences, a periplasmic flagellar system and a large proportion of genes of unknown function [3, 4, 20–24]. Evading the host’s innate immune system is critical during the early phase of infection, allowing leptospires to avoid complement‐mediated lysis, capture by neutrophil extracellular traps (NETs), phagocytosis and antimicrobial peptides (AMPs) [25–28]. Before reaching target tissues such as the kidneys or reproductive tract, leptospires must adjust rapidly to the host environment. Outer membrane proteins (OMPs) likely play key roles in this early host response, although many remain poorly characterised [21]. Several studies have shown that gene expression in *Leptospira* is modulated by host‐like conditions, such as increased temperature, osmolarity, iron limitation and serum exposure—factors that are all critical during host entry [29–32].

To study *Leptospira* under in vivo‐like conditions, a dialysis membrane chamber (DMC) model has been developed in which leptospires are cultivated in the peritoneal cavities of rats [33, 34]. This model has enabled transcriptional profiling of *L. interrogans* sv. Copenhageni in response to host signals. In our study, we employed the DMC model using sheep—a natural reservoir host—to examine the transcriptomic response of *L. interrogans* sv. Hardjo. While the peritoneal cavity is not a natural site of infection for *Leptospira* species, experimental inoculation of several pathogenic strains via this route allows them to survive and subsequently disseminate within host tissues [3].

Given the genetic differences between *L. interrogans* sv. Hardjo and *L. borgpetersenii* sv. Hardjo, we aimed to compare their early survival in the host environment. Although both species exhibit similar reservoir adaptation and cause largely indistinguishable clinical disease, our model showed that *L. borgpetersenii* sv. Hardjo was unable to survive in the sheep peritoneal cavity for 24 h. In contrast, *L. interrogans* sv. Hardjo was able to persist during this initial phase, enabling us to perform transcriptomic profiling under host‐like conditions. While not reflecting the natural route of infection, the model provides a useful approximation of the host environment during early colonisation. A better understanding of how *L. interrogans* sv. Hardjo responds to such conditions, supported by transcriptomic data and in vitro assays with defined host‐derived signals, may offer insights into mechanisms that support early survival and persistence in the cattle and sheep hosts.

## 2. Materials and Methods

### 2.1. Animals

Twenty‐four Polish long‐wool ewes, aged 1–2 years, were used in the study. To rule out prior infection or vaccination, the microscopic agglutination test (MAT) was conducted using *Leptospira* serovars (Bratislava, Canicola, Copenhageni, Grippotyphosa, Hardjo, Icterohaemorrhagiae and Pomona). Serum samples were tested using dilution patterns proposed by Ellis and Micha [35] from 1:10 to 1:30,000. All sheep tested negative. The experiments were conducted following the International Guidelines for Biomedical Research Involving Animals, approved by the 2nd Local Animal Care and Use Committee in Krakow, Poland (Committee Resolution Number 22/2021).

### 2.2. Bacterial Strains and Culture Conditions

To obtain *Leptospira* strains in a mammalian‐adapted state, *Leptospira* isolates were used in the experiments: *L. interrogans* sv. Hardjo (strains KR40, N116) and *L. borgpetersenii* sv. Hardjo (strains KR39, 58K3; Table 1). Bacteria were grown in T80/40/LH culture medium, used in bacteriological isolation of sv. Hardjo [36], at 28°C under aerobic conditions for 4 days until mid‐exponential phase, then counted using dark‐field microscopy. For animal studies, the bacterial density was adjusted to 10^6^/10^7^ leptospires/mL. Cultures underwent no more than three passages before being used in experiments.

**Table 1:** List of *Leptospira* strains used in the study.

Species	Serogroup	Serovar	Strain	Source
*L. interrogans*	Sejroe	Hardjo	KR40	Horse
*L. interrogans*	Sejroe	Hardjo	N116	Cattle
*L. borgpetersenii*	Sejroe	Hardjo	KR39	Cattle
*L. borgpetersenii*	Sejroe	Hardjo	58K3	Cattle

### 2.3. Implantation of Bacteria Culture in Peritoneal Cavity of Sheep

To culture *Leptospira* in a mammalian‐adapted state, organisms were grown in DMCs made from Spectra‐Por dialysis tubing (8000 Da MW cutoff) as previously described [37]. The sealed chambers were sterilised by boiling in 5 mM EDTA (Sigma–Aldrich) for 20 min, followed by two water‐only washes. After cooling, each DMC was filled with 100 mL ML medium containing 10% bovine serum albumin and 10^6^ organisms/mL. The DMCs were implanted into the sheep’s peritoneal cavity under anaesthesia (medetomidine 0.02 mg/kg, midazolam 0.3 mg/kg and buprenorphine 0.05 mg/kg). For each strain, DMCs were implanted into three different animals, which served as biological replicates. After 24 h, the sheep were euthanised with pentobarbital, DMCs were removed, and *Leptospira* counts were performed immediately using Petroff–Hauser chambers and flow cytometry. Live cells were quantified using reducing capacity and differential staining methods. The remaining membrane contents were immediately centrifuged to separate the bacterial pellet from the supernatant. The pellet was used for RNA isolation, while the remaining clarified supernatant was stored for subsequent protein‐based analyses, including defensin detection.

### 2.4. Viability and Quantification Tests for Leptospira spp.

The viability and quantification of *Leptospira* spp. were evaluated using the Alamar Blue assay and the LIVE/DEAD BacLight Bacterial Viability and Counting Kit (Invitrogen, Cat. Number L7012), following established protocols [38]. *Leptospira* cultures were plated in 96‐well plates with 180 µL of medium before and after DMC incubation. To assess viability, 20 µL of Alamar Blue was added, and plates were incubated for 72 h at 30°C. Metabolic activity was indicated by a colour change, and absorbance at 570 and 600 nm quantified resazurin reduction, reflecting bacterial viability. Heat‐killed leptospires (30 min at 100°C) were used as negative controls to confirm loss of viability. For further evaluation of bacterial viability and quantification, *Leptospira* cells were stained using the LIVE/DEAD BacLight Kit, which employs Syto9 to label all cells (live and dead) and propidium iodide (PI) to selectively label cells with compromised membranes. Stained cells were vortexed, incubated for 3 min in the dark, and analysed using flow cytometry or microscopy. A 5 µL aliquot was placed on a glass slide, covered with a coverslip, and imaged using fluorescence microscopy with appropriate excitation filters (Syto9:480/500, PI: 490/635 nm). Flow cytometry analysis was performed using the BD Accuri C6 (BD Biosciences) as outlined by Fontana et al. [39]. PI‐positive dead bacteria were detected on the FL3 channel, while Syto9‐positive live bacteria were measured on the FL1 channel. Control samples included live bacteria and heat‐inactivated bacteria (90°C) for comparison. Because *L. borgpetersenii* sv. Hardjo (KR39, 58K3) exhibited significantly reduced survival within DMCs, we conducted additional in vitro viability assays under stress conditions induced by various factors to model environmental conditions relevant to the peritoneal cavity. These experiments enabled a comparative evaluation of stress susceptibility across strains.

### 2.5. In Vitro Stress Exposure and Viability Assays

To investigate the susceptibility of *Leptospira* strains to environmental stressors relevant to the host peritoneal cavity, mid‐exponential‐phase cultures were subjected to controlled in vitro exposures. All experiments were conducted in T80/40/LH medium, and bacterial viability was assessed using the Alamar Blue assay, as described above. Viability was expressed as a percentage of resazurin reduction relative to the strain‐specific positive control.

To simulate osmotic and ionic shifts potentially encountered within the host peritoneal cavity, cultures were incubated in medium adjusted to ~ 300 mOsm/kg (+140 mM NaCl), 450 mOsm/kg (+200 mM NaCl) or 600 mOsm/kg (+300 mM NaCl) by supplementation with additional NaCl, or supplemented with KCl (150 mM), MgCl_2_ (20 mM), CaCl_2_ (20 mM), Na_2_SO_4_ (100 mM) or glucose (100 mM) at 37°C for 24 h. This setup was based on protocols reported by Matsunaga et al. [40] and Choy et al. [41]. The NaCl gradient was designed to mimic progressively increasing osmotic stress. KCl and Na_2_SO_4_ were included to assess the effects of specific ionic species under hypertonic conditions, while MgCl_2_ and CaCl_2_ concentrations were selected based on standard values used in bacterial osmotic stress studies. Glucose served as a non‐ionic osmolyte control.

Iron limitation was induced by supplementing T80/40/LH medium with 2,2^′^‐dipyridyl (Sigma–Aldrich) at final concentrations of 20 or 40 µg/mL. Cultures at mid‐exponential phase were incubated aerobically at 37°C for 72 h. Conditions were selected based on established iron‐restriction protocols in *Leptospira* by Lo et al. [42].

Oxidative stress was induced by exposing *Leptospira* cultures to hydrogen peroxide (H_2_O_2_) at final concentrations of 4, 10 µM or 1 mM for 1, 2 or 4 h at 30°C. Immediately after exposure, Alamar Blue was added, and metabolic activity was quantified spectrophotometrically at 570 and 600 nm. The protocol was adapted from previous studies on oxidative stress response in *Leptospira* spp [43, 44].

### 2.6. Dot Blot Analysis

Immediately after removal of the DMCs from the sheep peritoneal cavity, their contents were centrifuged to yield a clear supernatant used for defensin detection and a bacterial pellet reserved for transcriptomic analysis. For each DMC, three independent 1 mL aliquots of supernatant were collected and stored at −80°C. These aliquots were then lyophilised under vacuum for 24 h using a Benchtop 2K freeze dryer (Virtis), following protocols optimised for concentrating biological fluids such as peritoneal dialysate [45, 46]. The resulting dry pellet was stored at −80°C until further processing. Immediately prior to dot‐blot, the lyophilised pellets were reconstituted in 30 µL TBST buffer (20 mM Tris–HCl, pH 7.6; 150 mM NaCl; 0.05% Tween‐20) and pooled to combine material originating from the same DMC. The pooled samples were then spotted (2 µL) onto a PVDF (Merck Millipore) and left to air‐dry (~30 min). Membranes were blocked for 1 h at room temperature in 1% BSA and incubated with primary antibodies against α‐defensin‐1 (FNab02326 /FineTest) and β‐defensin‐1 (FNab02327/FineTest), each diluted in 1% BSA (Merck Millipore), for 1 h. After three washes in TBST, membranes were incubated with secondary HRP‐conjugated goat anti‐mouse antibodies (1:3000, Vector) for 1 h, followed by another set of washes. Chemiluminescent detection was performed using ECL substrate; signal capture was done with the G:BOX Chemi system (Syngene; GeneSys/GeneTools). As a positive control, recombinant α‐defensin‐1 (Thermo Fisher Scientific, Cat. Number 300‐42) and recombinant β‐defensin‐1 (Thermo Fisher Scientific, Cat. Number 300–51) were spotted on the same membrane under identical conditions.

### 2.7. RNA Isolation and RNA‐Seq Library Preparation

For each strain, in vitro bacterial cultures were prepared in triplicate to account for technical variation. Similarly, bacteria from in vivo cultures came from three different animals being biological replicates. The collected cultures/fluids were centrifuged for 40 min in 4500 rpm to pellet bacteria. Bacterial pellets were used for total RNA isolation using TRI Reagent Solution (ThermoFisher) according to the manufacturer’s protocol. Integrity of the isolated RNA was analysed using TapeStation RNA ScreenTapes (Agilent Technologies), and only samples with RIN >7 were used for further analysis. 5 µg of the purified total RNA was used as an input for ribosomal RNA depletion with Ribo‐off rRNA Depletion Kit V2 (Bacteria) and subsequent library preparation with VAHTS Universal V6 RNA‐seq Library Prep Kit for Illumina (Vazyme). The resulting libraries were controlled for quality by TapeStation D1000 ScreenTape (Agilent Technologies) and quantified with the Qubit dsDNA BR kit (ThermoFisher). An equimolar pool of libraries was commercially sequenced in a 150 bp paired‐end run at the Oklahoma Medical Research Foundation NGS Core (USA) to obtain more than 8 million (M) reads per sample.

### 2.8. Data Analysis

Raw sequencing reads were controlled for quality using FastQC software (https://www.bioinformatics.babraham.ac.uk/projects/fastqc/) and filtered/trimmed using Flexbar [47] software. The filtered reads were aligned to the corresponding genomes (KR40 NCBI RefSeq accession GCF_023158895.1; N116 NCBI RefSeq accession: GCF_023515975.1) [18]. The mapping procedure was done using a modified procedure in Bowtie2 [48] software. The modification included the very sensitive mode and keeping only a single best genome alignment. The mapped reads were counted using the HTSeq‐count [49] procedure. Orthologue clustering between the two Hardjo strains was conducted using the GET_HOMOLOGUES software, employing the OrthoMCL algorithm with a 45% sequence identity threshold [50]. Subsequently, differential expression analysis of the set of shared orthologous genes was performed using read counts and the DESeq2 software [51]. Gene annotation was based on the KR40 strain. Genes with an adjusted *p*‐value (false discovery rate, FDR) < 0.05, calculated using the Benjamini–Hochberg procedure [52] were considered as differentially expressed genes (DEGs). To explore the variability of the data, principal component analysis (PCA) and a heatmap were performed and visualised using ClustVis [53].

In addition, using genome data from *L. borgpetersenii* sv. Hardjo strains KR39 (BioProject: PRJNA828006), 58K3 (BioProject: PRJNA828000), LB197 (BioProject: PRJNA16148) and 203 (BioProject: PRJNA384237), a comparative analysis was performed in GET_HOMOLOGUES (45% sequence identity threshold) to identify proteins specific to *L. interrogans* sv. Hardjo, defined as those present in both KR40 and N116 genomes and lacking orthologues across the of the representative *L. borgpetersenii* sv. Hardjo genomes.

To investigate gene functions and associated pathways, protein‐coding sequences were annotated to Clusters of Orthologous Genes (COG) categories [54], using eggNOG‐mapper v2 [55] (parameters: ‐e‐value 0.01 ‐score 50 ‐pident 40 ‐query_cover 20 ‐subject_cover 20 ‐target_orthologs all). For each category, the proportion of DEGs was calculated relative to the total number of genes assigned to that category. Sequences were also cross‐referenced in UniProt [56]. Selected genes were manually curated from the literature with similarity searches performed with BLASTp at ≥45% sequence identity [57]. Operons were predicted using Operon‐mapper [58]. Only genes fulfilling both criteria (FDR < 0.05 and |log_2_FC| > 1) are presented in the main‐text tables, whereas the complete list of DEGs is provided in the Supporting Information. Genes are ordered by ascending FDR value.

### 2.9. Confirmation of DEGs by Quantitative RT‐PCR

About 15 mL of *Leptospira* cultures from iron limitation and oxidative stress experiments were used for total RNA isolation as described above. RNA quality and yield were checked via the A260:280 ratio using a NanoDrop spectrophotometer. cDNA was synthesised using the High‐Capacity cDNA Reverse Transcription Kit (Applied Biosystems). Gene expression analysis was carried out using primers designed with Primer3Plus [59], as listed in Supporting Information 1: Table S1, using the LightCycler 96 System (Roche) with SYBR Green master mix (Applied Biosystems). Amplification specificity was confirmed through melting curve analysis and agarose gel electrophoresis. Gene expression was normalised to reference genes *16S rRNA* using the 2^−ΔΔCt^ method.

## 3. Results

### 3.1. Effect of Host–Bacteria Interactions on the Viability of Leptospira

Initially, four laboratory‐adapted sv. Hardjo strains, belonging to two distinct *Leptospira* species (*L. interrogans*: KR40, N116; *L. borgpetersenii*: KR39, 58K3), were cultured and introduced into the peritoneal cavities of sheep using DMCs. This experimental setup aimed to investigate potential variations in host–bacteria interactions among different species within the same serovar. Analysis of bacterial cultures using the LIVE/DEAD BacLight Bacterial Viability and Counting Kit (Figure 1A), together with the Alamar Blue viability assay (Figure 1B), demonstrated that *L. borgpetersenii* sv. Hardjo strains (KR39, 58K3) did not survive after 24 h of incubation in DMCs implanted in the peritoneal cavities of sheep. In contrast, *L. interrogans* sv. Hardjo strains (KR40, N116) exhibited survival for 24 h. The number of bacteria surviving within the DMC increased with the incubation time (Supporting Information 2: Table S2). All samples contained actively moving spirochaetes, clearly visible under dark‐field microscopy.

Figure 1Survival of *Leptospira* spp. after 24 h of incubation in DMC implanted into the ovine peritoneal cavity. (A) Bacterial viability was assessed using LIVE/DEAD BacLight staining and analysed by flow cytometry, with representative fluorescence and dark‐field microscopy images (scale bar: 10 µm). (B) Metabolic activity was measured using the Alamar Blue assay. Data are presented as mean ± SD of technical triplicates from three biological replicates.

**Figure:**
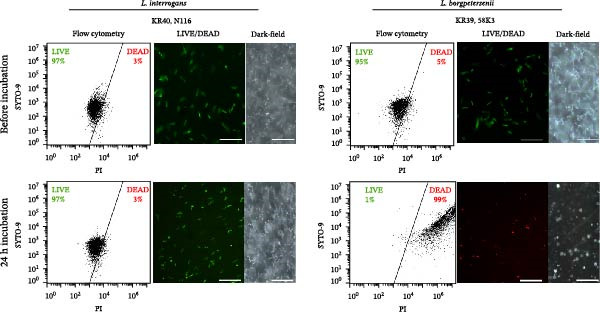
(A)

**Figure:**
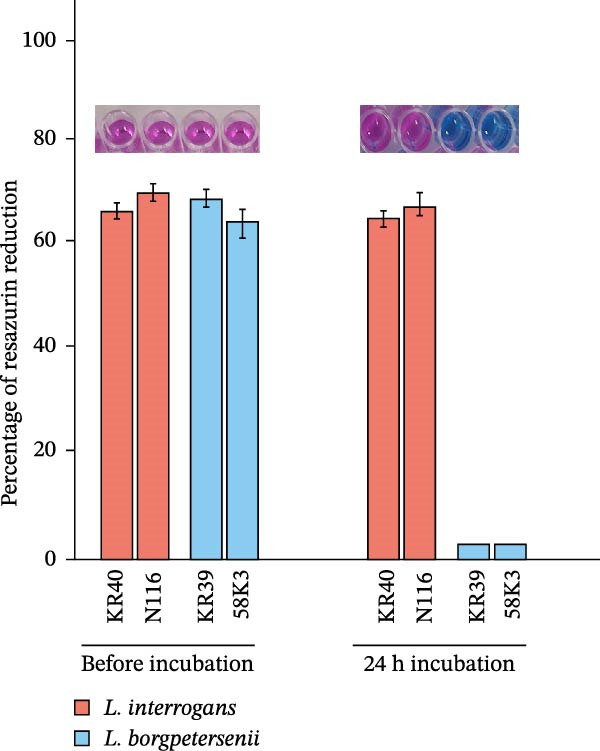
(B)

### 3.2. Viability of Leptospira Strains Under Osmotic, Oxidative and Iron‐Limiting Conditions

To assess whether abiotic factors present in the peritoneal cavity could account for the poor recovery of *L. borgpetersenii* strains in the DMC model, all four study strains were tested in vitro under stress conditions mimicking iron limitation, oxidative stress and osmotic/ionic environments. Viability was measured using the Alamar Blue assay and is expressed as a percentage of resazurin reduction relative to the strain‐specific control (Figure 2).

Figure 2In vitro viability of *Leptospira* strains under stress conditions mimicking the peritoneal environment. Viability was assessed by the Alamar Blue assay and expressed as a percentage relative to the strain‐specific positive control (Ctrl) under iron limitation (A), oxidative stress (B) and osmotic and ionic stress (C). Representative images of resazurin‐stained cultures are shown above each panel. Bars represent mean ± SD from nine biological replicates. Asterisks indicate significant differences compared with controls ( ^∗^ *p* < 0.05,  ^∗∗^ *p* < 0.01,  ^∗∗∗^ *p* < 0.001).

**Figure:**
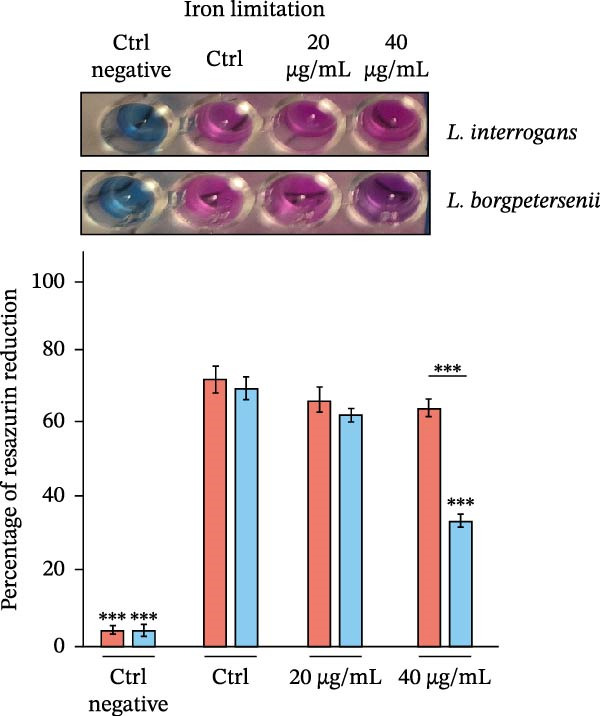
(A)

**Figure:**
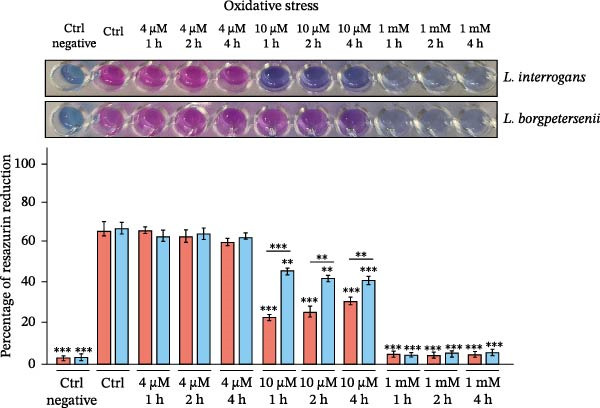
(B)

**Figure:**
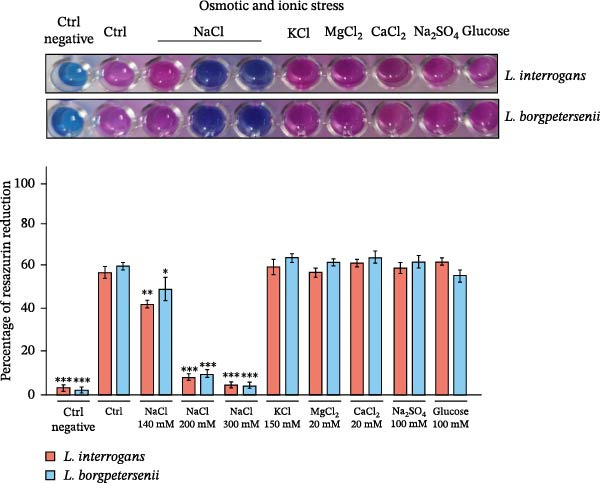
(C)

Under iron‐limiting conditions (Figure 2A), mild iron restriction caused a moderate decrease in metabolic activity across all strains. At higher 2,2^′^‐dipyridyl concentrations, *L. borgpetersenii* sv. Hardjo strains showed markedly reduced viability (~33%–37% of control), whereas *L. interrogans* sv. Hardjo retained over 60% metabolic activity.

During oxidative stress (Figure 2B), low concentrations of H_2_O_2_ had little effect on viability. At 10 µM, *L. borgpetersenii* sv. Hardjo maintained higher residual activity (~50%) than *L. interrogans* sv. Hardjo (~40%), while exposure to 1 mM H_2_O_2_ resulted in near‐complete loss of viability in all strains.

Under osmotic stress (Figure 2C), increasing NaCl concentration led to a progressive decline in viability. At 200 mM NaCl, *L. borgpetersenii* strains dropped below 25% metabolic activity, whereas *L. interrogans* strains retained ~45%. Alternative ionic or non‐ionic supplements did not significantly affect viability.

Overall, *L. borgpetersenii* sv. Hardjo displayed increased susceptibility to elevated osmolarity and severe iron depletion, while their tolerance to sublethal oxidative stress was higher than that of *L. interrogans* sv. Hardjo.

### 3.3. Detection of Host Defensins in DMC Supernatants

In order to determine whether host antimicrobial factors could enter the chambers, we analysed DMC fluids for α‐ and β‐defensins. No α‐ or β‐defensin signal was detected in the negative controls, including sterile culture medium, native peritoneal fluid collected before DMC implantation or fluid from DMCs incubated with medium alone without bacteria (Supporting Information 3: Figure S3). In contrast, α‐ and β‐defensins were readily detected in supernatants from DMCs containing leptospiral strains, indicating that host defensins had penetrated the membrane into the fluid inside the DMC. The strongest signals, both for α‐ and β‐defensins, were observed for *L. borgpetersenii* strains KR39 and 58K3, with moderately weaker signal for KR40 and N116. The strains that triggered the strongest defensin signal—KR39 and 58K3—did not survive 24 h of in vivo incubation.

### 3.4. RNA Sequencing and Quality Control

To characterise the early transcriptional adjustments triggered during host entry, RNA isolated from *Leptospira* cells that remained viable after 24 h in vivo (DMC) and from in vitro cultures was subjected to RNA‐seq analysis. For each strain, six RNA samples were analysed, comprising three biological replicates from the in vivo DMC condition and three in vitro control cultures. For strain KR40, a total of 45.11M raw reads were obtained (7.51 million per sample), of which 96.04% on average passed initial filtering. Of the filtered reads, 98.88% were mapped to the reference genome. Similarly, for strain N116, 55.42M raw reads were obtained (9.23M per sample), with 96.71% on average passing quality filtering and 99.19% of the filtered reads successfully mapped to the reference genome (Supporting Information 4: Table S4). PCA on the set of genes shared between strains KR40 and N116 clearly separated the samples into two distinct clusters corresponding to in vitro control and the 24 h in vivo DMC condition (Figure 3A). The heatmap of DEGs further showed that sample clustering was driven predominantly by experimental condition (Figure 3B). While the two strains remained separated in the in vitro group, after 24 h in vivo some replicate data from both strains intermingled and clustered together.

Figure 3Global transcriptomic profiles of *L. interrogans* sv. Hardjo under in vivo and in vitro conditions. (A) PCA shows separation of samples recovered after 24 h in vivo in the DMC model and in vitro culture; each point represents a replicate, the symbol denotes the strain and ellipses indicate 95% confidence intervals. (B) Hierarchical clustering heatmap of differentially expressed genes, showing relative expression levels (blue—lower, red—higher). Rows represent genes, columns represent biological replicates; coloured bars indicate condition and strain.

**Figure:**
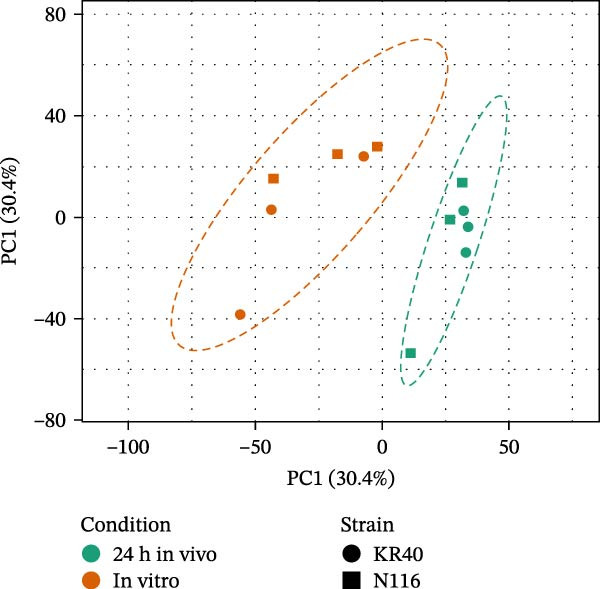
(A)

**Figure:**
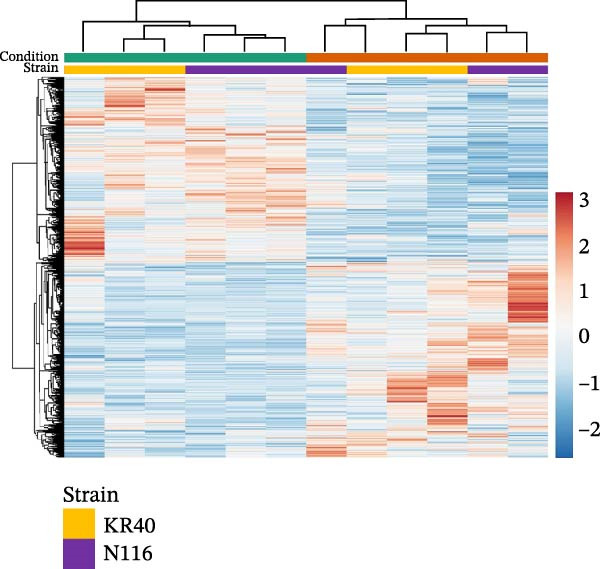
(B)

### 3.5. Early Transcriptional Reprogramming Under Host Conditions

**Figure 4:**
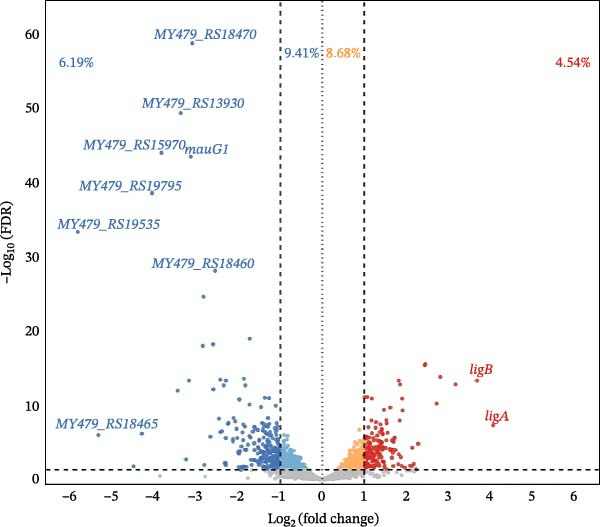
Volcano plot of differentially expressed genes in *Leptospira interrogans* sv. Hardjo after 24 h in the DMC model compared with in vitro conditions. Red and blue dots represent significantly (FDR < 0.05) upregulated or downregulated genes, respectively; grey dots correspond to non‐significant changes. Genes with |log_2_(FoldChange)| > 1 are depicted with more intense colours. Most differentially expressed genes are annotated with their identifier.

Early transcriptional responses were next examined by comparing the gene expression profiles of *L. interrogans* sv. Hardjo grown in vitro with those recovered after 24 h of in vivo incubation within the DMC model. Two distinct strains (KR40, N116) belonging to the same species and serovar were selected for this experiment to ensure that the observed transcriptional changes reflect conserved host adaptation mechanisms rather than strain‐specific responses. The two strains share a core genome of 3698 genes. A total of 1066 genes (29% of total genes) were found to be significantly altered under host conditions (FDR < 0.05; Figure 4), of which 305 (28.9%) were not represented by orthologues in *L. borgpetersenii* sv. Hardjo. Among DEGs, 459 (44.3%) genes were upregulated, while 577 (55.7%) were downregulated. The majority of genes exhibited only slight transcriptional shifts, within an absolute log_2_FC range of 0–1, indicating a balanced transcriptional adjustment occurring within the first 24 h of host exposure. The most pronounced upregulation was detected for *ligA* (log_2_FC = 4.07), whereas the most strongly downregulated gene was *MY479_RS19535*, encoding a sulphatesulfate transport anti‐sigma (STAS) domain‐containing protein (log_2_FC = –5.84). A complete list of all DEGs, ordered separately for upregulated and downregulated transcripts and sorted by ascending FDR values, is provided in Supporting Information 5: File S5. For each gene, the file also indicates whether the corresponding locus lacks orthologues in representatives of *L. borgpetersenii* sv. Hardjo.

To obtain a more biologically meaningful subset and reduce the influence of low‐amplitude transcriptional fluctuations, we subsequently focused on genes that met both significance and fold‐change thresholds (FDR < 0.05; |log_2_FC| > 1; Figure 4). This more stringent criterion highlights genes exhibiting robust and potentially functionally relevant transcriptional responses to host conditions. In total, 397 genes fulfilled these criteria, including 168 (4.54%) upregulated and 229 (6.19%) downregulated genes. Of these upregulated genes, 71 (42.3%) encoded proteins without orthologues in the *L. borgpetersenii* sv. Hardjo, while 91 (39.7%) of the downregulated genes lacked orthologues in this species.

#### 3.5.1. Functional Categorisation of DEGs

**Figure 5:**
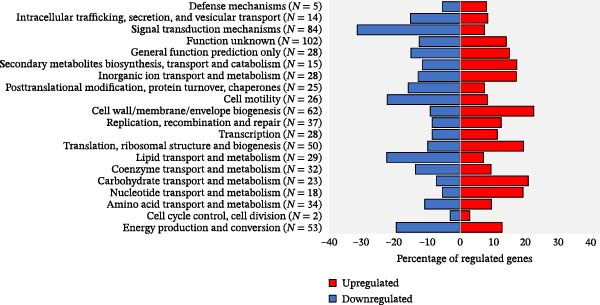
COG category distribution of differentially expressed genes in *L. interrogans* sv. Hardjo after 24 h incubation in the DMC model compared with in vitro conditions. Each bar represents a COG functional category. Red indicates the proportion of upregulated genes and blue the proportion of downregulated genes (FDR < 0.05). Values in parentheses indicate the total number of differentially expressed genes per category.

Overall, ~61% of the *L. interrogans* sv. Hardjo genome could be assigned to Clusters of Orthologous Groups functional categories. Within this framework, 369 downregulated (64% of all downregulated genes) and 294 upregulated DEGs (64% of all upregulated genes) were assigned to COG categories (Figure 5), with some genes classified into more than one category. A complete listing of all DEGs and their corresponding COG category assignments is provided in Supporting Information 5: File S5.

Among the upregulated genes, notable changes were observed in categories related to the bacterial genetic machinery and protein synthesis (‘Translation, ribosomal structure and biogenesis,’ ‘Transcription,’ ‘Replication, recombination and repair’), highlighting the activation of core biosynthetic processes required for rapid adjustment to the host environment. Furthermore, a considerable proportion of upregulated genes belonged to ‘Cell wall/membrane/envelope biogenesis,’ consistent with active surface remodelling. Among the downregulated genes, the most represented functional categories were ‘Signal transduction mechanisms’ and ‘Cell motility.’ Several metabolism‐related categories also displayed differential regulation, with ‘Carbohydrate transport and metabolism’ mainly upregulated, and ‘Lipid transport and metabolism’ predominantly downregulated. These changes were accompanied by alterations in ‘Energy production and conversion,’ indicating broad metabolic reorganisation.

Since many genes were classified as ‘Function unknown’ or lacked identifiable orthologues in the COG database, a literature‐based annotation was undertaken to better characterise the major pathways. Given the observed differences in bacterial survival under in vitro conditions and differences in the abundance of defensins in the DMC supernatants, additional focus was placed on genes related to adhesion, lipoproteins, stress response and iron‐related genes, as these pathways may potentially explain the survival of *L. interrogans* sv. Hardjo but not *L. borgpetersenii* sv. Hardjo under the same experimental DMC conditions.

#### 3.5.2. Differential Expression of Membrane‐Associated Genes, Including Lipoproteins and Putative Adhesins

As many DEGs were assigned to the ‘Cell wall/membrane/envelope biogenesis’ category, we next examined proteins localised to these compartments. These proteins are known to play key roles in environmental sensing and host interaction, including adhesion [20]. Among these, lipoproteins represented a substantial subgroup (Table 2, Supporting Information 6: Table S6). Most of the genes were predicted to have no orthologues in *L. borgpetersenii* sv. Hardjo.

**Table 2:** Selected differentially expressed membrane‐associated genes of *L. interrogans* sv. Hardjo after 24 h in the ovine DMC model.

ORF ID^a^	Gene^a^	Product^b^	Log_2_FC^c^	FDR^d^	Absent in LbH^e^
Upregulated					
* MY479_RS08695*	—	Lipoprotein	2.45	2.88E‐16	—
* MY479_RS02460*	*ligB*	Lipoprotein adhesin LigB	3.69	5.09E‐14	—
* MY479_RS00790*	*yidC*	Membrane protein insertase YidC	1.38	4.13E‐08	—
* MY479_RS00455*	—	Lipoprotein	1.53	1.04E‐08	Yes
* MY479_RS02465*	*ligA*	Lipoprotein adhesin LigA	4.07	6.7E‐08	Yes
* MY479_RS01835*	*lolA*	Outer‐membrane lipoprotein carrier protein LolA	1.02	1.92E‐06	—
* MY479_RS00785*	*yidD*	Membrane protein insertion efficiency factor YidD	1.72	2.50E‐06	—
* MY479_RS08840*	*mce*	Mammalian cell entry protein Mce	1.02	3.77E‐06	—
* MY479_RS16695*	*lenF*	Endostatin‐like outer membrane lipoprotein LenF	1.38	8.07E‐06	Yes
* MY479_RS10850*	*lmtA*	Lipid A Kdo2 1‐phosphate O‐methyltransferase	1.14	1.45E‐05	—
* MY479_RS13150*	—	LIC10774 family surface protein/DUF1565 domain‐containing protein	1.44	4.78E‐05	Yes
* MY479_RS01900*	—	SBBP repeat beta‐propeller lipoprotein, LipL53 family	1.62	1.11E‐04	Yes
* MY479_RS01875*	*lsa21*	Lipoprotein Lsa21	1.74	1.12E‐04	Yes
* MY479_RS15505*	*lenC*	Endostatin‐like outer membrane lipoprotein LenC	1.08	2.13E‐04	Yes
* MY479_RS15790*	—	SBBP repeat beta‐propeller lipoprotein, LipL53 family	1.68	2.65E‐04	—
* MY479_RS07110*	—	SBBP repeat beta‐propeller lipoprotein, LipL53 family	1.37	4.06E‐04	—
* MY479_RS04310*	—	Collagenase	1.00	2.19E‐03	—
* MY479_RS01890*	—	SBBP repeat beta‐propeller lipoprotein, LipL53 family	1.29	2.82E‐03	Yes
* MY479_RS02610*	—	Lipoprotein (LipL46 like)	1.01	7.51E‐03	Yes
Downregulated					
* MY479_RS13930*	—	DUF1566 domain‐containing protein	−3.38	4.98E‐50	—
* MY479_RS15970*	—	SbsA Ig‐like domain‐containing protein	−3.84	1.17E‐44	Yes
* MY479_RS19795*	—	RHS repeat‐associated core domain‐containing protein	−4.06	3.12E‐39	Yes
* MY479_RS19790*	—	Lipoprotein	−3.18	5.09E‐14	Yes
* MY479_RS15785*	*lipL36*	Lipoprotein LipL36	−1.60	1.56E‐07	—
* MY479_RS00190*	—	Lipoprotein	−1.50	4.73E‐06	Yes
* MY479_RS15810*	—	Lipoprotein	−1.27	1,05E‐05	Yes
* MY479_RS06440*	—	Lipoprotein	−1.77	2.14E‐05	Yes
* MY479_RS10440*	*gspC*	General secretion pathway protein GspC	−1.16	1.66E‐04	—
* MY479_RS01865*	—	DUF1565 domain‐containing protein	−1.70	2.20E‐03	Yes
* MY479_RS01610*	—	LA_0364 family Cys‐rich lipoprotein	−1.16	5.72E‐03	Yes
* MY479_RS11510*	*lipL32*	Major surface lipoprotein LipL32	−1.09	1.12E‐02	—
* MY479_RS14800*	—	TIGR04452 family lipoprotein	−1.69	1.15E‐02	Yes
* MY479_RS03150*	—	Lipoprotein	−1.94	4.80E‐02	Yes

^a^Gene identifiers based on NCBI RefSeq annotation of *L. interrogans* sv. Hardjo strain KR40 (GCF_023158895.1).

^b^Description based on combined RefSeq and UniProt annotations.

^c^Log_2_ fold change between in vivo and in vitro conditions.

^d^False discovery rate.

^e^Genes without orthologues in *L. borgpetersenii* sv. Hardjo.

Among the most strongly upregulated were genes encoding the lipoprotein adhesins *ligA* and *ligB*, both previously associated with host extracellular matrix interaction and immune modulation [41]. Similarly, *lenF* and *lenC*, encoding the endostatin‐like outer membrane lipoproteins [60], were significantly upregulated. Previous analyses have shown that *ligA*, *lenF*, *lenC* are absent in *L. borgpetersenii*, whereas *ligB* is conserved [12]. In addition to these well‐characterised adhesins, several other upregulated genes have also been implicated in adhesion or host interaction. Among them was *lsa21*, as well as four genes with similarity to *lipL53*. Additional upregulated factors included *mce*, encoding the mammalian cell entry protein [61], *MY479_RS04310* encoding a collagenase [62], or *MY479_RS13150*, described as a surface‐exposed calcium‐binding protein gene with a possible role in adhesion [63].

Conversely, several genes were downregulated, most notably *MY479_RS13930*, encoding a putative OMP. Its orthologue in *L. borgpetersenii* sv. Hardjo (*lbl2618*) has been proposed to act as a surface adhesin capable of binding extracellular matrix components such as fibronectin and fibrinogen [64]. Phylogenetic analysis has shown substantial variability among these gene, suggesting possible host‐specific adaptation. Other downregulated lipoprotein genes included *lipL36*, consistent with earlier reports that its expression is reduced during mammalian infection [65]. The *lipL32*, which is generally described as highly expressed under both in vitro and in vivo conditions [66], was downregulated in our study.

Several genes directly involved in membrane remodelling were also differentially expressed. The type II secretion system (T2SS) was slightly downregulated despite the pronounced shifts observed in lipoprotein expression (Supporting Information 6: Table S6). Affected genes included *gspN*, *gspK*, *gspJ*, *gspE* and *gspC*. In contrast, upregulated genes included those related to membrane protein insertion, such as *yidC* or *yidD* which in other bacteria enable the integration and folding of membrane proteins [67], as well as lipoprotein carrier *lolA*, a periplasmic carrier responsible for shuttling lipoproteins to the outer membrane.

Minor alterations were noted in lipopolysaccharide biosynthesis, including slight downregulation of *lpxA*, *lpxK* and the LPS exporter component *lptB* (Supporting Information 6: Table S6). In contrast, *lmtA*, involved in lipid A modification, was upregulated, together with several genes associated with O‐antigen assembly, as discussed in the section on carbohydrate metabolism (Table 3).

**Table 3:** Selected differentially expressed energy and metabolism genes of *L. interrogans* sv. Hardjo after 24 h in the ovine DMC model.

ORF ID^a^	Gene^b^	Product^b^	Log2FC^c^	FDR^d^	Absent in LbH^e^
Upregulated					
* MY479_RS15425*	*sul1*	Carbonic anhydrase	3.18	1.72E‐13	—
* MY479_RS19055*	—	Glycosyltransferase RgtA/B/C/D‐like domain‐containing protein	1.41	1.91E‐07	—
* MY479_RS12025*	*atpH*	ATP synthase F1 subunit delta	1.13	2.39E‐06	—
* MY479_RS12035*	*atpE*	ATP synthase F0 subunit C	1.23	5.91E‐06	—
* MY479_RS12030*	*atpF*	F0F1 ATP synthase subunit B	1.10	9.11E‐06	—
* MY479_RS04540*	—	Glycosyltransferase family 4 protein	1.14	1.15E‐04	—
* MY479_RS01635*	*rpiB*	Ribose 5‐phosphate isomerase B	1.06	7.50E‐04	—
* MY479_RS19205*	*alr*	Alanine racemase	1.01	9.40E‐04	—
* MY479_RS06065*	*glnA*	Glutamine synthetase	1.12	1.12E‐03	—
* MY479_RS09620*	*fbp*	Class 1 fructose‐bisphosphatase	1.41	1.03E‐03	—
* MY479_RS16575*	*uphC*	Glycerol‐3‐phosphate antiporter	1.20	1.53E‐03	—
* MY479_RS12040*	*atpB*	F0F1 ATP synthase subunit A	1.05	2.28E‐03	—
* MY479_RS01895*	—	Glycosyltransferase RgtA/B/C/D‐like domain‐containing protein	1.23	2.45E‐03	Yes
* MY479_RS06535*	*sch*	Beta‐ketoacyl synthase	1.03	8.04E‐03	—
* MY479_RS06505*	*fabH*	3‐oxoacyl‐[acyl‐carrier‐protein] synthase	1.01	1.26E‐02	—
Downregulated					
* MY479_RS15970*	—	Nucleoside‐diphosphate sugar epimerase	−3.84	1.17E‐44	Yes
* MY479_RS01855*	*ivd*	Isovaleryl‐CoA dehydrogenase	−1.41	3.09E‐08	—
* MY479_RS00195*	—	GMC oxidoreductase	−1.40	9.78E‐08	Yes
* MY479_RS09950*	*glpA2*	Glycerol‐3‐phosphate dehydrogenase	−1.42	2.57E‐07	Yes
* MY479_RS09390*	—	Glycosyltransferase	−1.66	3.65E‐07	—
* MY479_RS04530*	—	VOC family protein	−1.30	4.79E‐06	Yes
* MY479_RS04635*	*trpA*	Tryptophan synthase subunit alpha	−1.05	6.02E‐06	—
* MY479_RS16415*	—	Alpha/beta fold hydrolase	−1.63	1.40E‐05	Yes
* MY479_RS05255*	*gspA*	1‐acyl‐sn‐glycerol‐3‐phosphate acyltransferase	−1.05	1.79E‐05	—
* MY479_RS03035*	*caiA1*	Acyl‐CoA dehydrogenase	−1.01	2.89E‐05	—
* MY479_RS17495*	*acs*	Acetate—CoA ligase	−1.13	4.82E‐05	—
* MY479_RS00380*	*dapF*	Diaminopimelate epimerase	−1.13	8.20E‐05	—
* MY479_RS16420*	—	Cholesterol oxidase	−1.31	1.04E‐04	Yes
* MY479_RS00285*	*cysE*	Serine O‐acetyltransferase	−1.02	1.63E‐04	—
* MY479_RS10970*	—	Alpha/beta hydrolase	−1.32	4.63E‐04	Yes
* MY479_RS15545*	*hisI*	Phosphoribosyl‐AMP cyclohydrolase	−1.41	1.21E‐03	—
* MY479_RS15395*	—	Lactonizing lipase	−3.26	1.92E‐03	Yes
* MY479_RS12370*	*lpdA*	Dihydrolipoyl dehydrogenase (E3)	−1.15	5.10E‐03	—
* MY479_RS02440*	—	VOC family protein	−1.08	1.53E‐02	Yes
* MY479_RS08630*	*acoB*	Pyruvate dehydrogenase complex E1 component subunit beta	−1.22	1.57E‐02	—

^a^Gene identifiers based on NCBI RefSeq annotation of *L. interrogans* sv. Hardjo strain KR40 (GCF_023158895.1).

^b^Description based on combined RefSeq and UniProt annotations.

^c^log_2_ fold change between in vivo and in vitro conditions.

^d^False discovery rate.

^e^Genes without orthologues in *L. borgpetersenii* sv. Hardjo.

#### 3.5.3. Differential Expression of Iron‐Related Genes

Given the reduced survival of *L. borgpetersenii* sv. Hardjo under in vitro iron limitation conditions compared with *L. interrogans* sv. Hardjo, particular attention was directed towards proteins previously reported as iron‐regulated [42, 68]. Selected iron‐related DEGs are shown in Table 4, whereas the complete list is provided in Supporting Information 6: Table S6.

**Table 4:** Selected differentially expressed iron‐related genes of *L. interrogans* sv. Hardjo after 24 h in the ovine DMC model.

ORF ID^a^	Gene^b^	Product^b^	Log2FC^c^	FDR^d^	Absent in LbH^e^
Upregulated					
* MY479_RS08695*	—	Lipoprotein	2.45	2.88E‐16	—
* MY479_RS13360*	—	Hypothetical protein	2.81	1.61E‐14	Yes
* MY479_RS13365*	—	TonB‐dependent receptor	1.91	1.45E‐11	—
* MY479_RS14660*	—	Di‐haem oxidoredictase family protein	1.62	2.18E‐10	—
* MY479_RS14655*	*lruB*	Imelysin LruB	1.92	5.39E‐10	—
* MY479_RS09695*	—	TonB‐dependent receptor	1.67	5.96E‐06	—
* MY479_RS14665*	—	Imelysin family protein	1.07	2.08E‐05	—
* MY479_RS11530*	—	TonB‐dependent receptor	1.12	9.89E‐05	Yes
* MY479_RS09690*	—	LIC_11695 family lipoprotein	1.82	4.50E‐04	Yes
* MY479_RS15895*	—	LruC domain‐containing protein	1.11	1.35E‐02	Yes
Downregulated					
* MY479_RS07010*	—	DUF3015 domain‐containing protein	−2.13	7.4E‐06	Yes
* MY479_RS15530*	—	DUF2339 domain‐containing protein	−1.15	7.10E‐04	Yes
* MY479_RS12095*	—	Iron dicitrate transport regulator FecR	−1.05	2.15E‐02	Yes
* MY479_RS12550*	—	FecR domain‐containing protein	−1.99	2.35E‐02	—

^a^Gene identifiers based on NCBI RefSeq annotation of *L. interrogans* sv. Hardjo strain KR40 (GCF_023158895.1).

^b^Description based on combined RefSeq and UniProt annotations.

^c^log_2_ fold change between in vivo and in vitro conditions.

^d^False discovery rate.

^e^Genes without orthologues in *L. borgpetersenii* sv. Hardjo.

Among these, two imelysin genes (*lruB* and *MY479_RS14665*) together with a di‐haem oxidoreductase (*MY479_RS14660*) were markedly upregulated and are likely co‐transcribed as part of a single operon [69]. Additionally, *MY479_RS15895*, encoding a lipoprotein with a LruC domain, was also upregulated. Notably, both LruB and LruC have been present in ocular fluids of horses with equine recurrent uveitis, where they elicit marked antibody responses [70, 71].

Genes possibly involved in iron acquisition also showed pronounced transcriptional changes, including several TonB‐dependent receptor (TBDR) genes, most notably: *MY479_RS09695* (putative desferrioxamine receptor), *MY479_RS13365* (putative hemin receptor) and *MY479_RS11530* (putative ferrichrome‐iron receptor, with no ortholog in *L. borpetersenii*). TBDRs are OMPs mediating the uptake of scarce nutrients such as iron [72]. Two TBDR genes are part of putative operons that include strongly upregulated proteins of unknown function (*MY479_RS13360-MY479_RS13370* and *MY479_RS09690-MY479_RS09695*), which lack orthologues in *L. borgpetersenii* sv. Hardjo. Notably, *MY479_RS13360* was upregulated in the *perRAperRB* double mutant [73], while *MY479_RS09690* was upregulated in iron‐limiting conditions [42]. Several *fecR*‐like genes showed downregulation, with the most pronounced changes observed for *MY479_RS12550 and MY479_RS12095*, the latter lacking an orthologue in *L. borgpetersenii*. In *E. coli* FecR functions as an anti‐σ factor of the FecI signalling cascade [74].

#### 3.5.4. Differential Expression of Stress and Redox Homeostasis Related Genes

As *L. borgpetersenii* sv. Hardjo shows greater tolerance to oxidative stress under in vitro conditions compared with *L. interrogans* sv. Hardjo, we next assessed whether stress‐related and redox‐balancing genes are activated in *L. interrogans* sv. Hardjo under in vivo conditions. Selected DEGs are presented in Table 5, while the complete list is provided in Supporting Information 6: Table S6. Most of these genes showed downregulation, with only a few lacking orthologues in *L. borgpetersenii* sv. Hardjo.

**Table 5:** Selected differentially expressed stress response and redox homeostasis genes of *L. interrogans* sv. Hardjo after 24 h in the ovine DMC model.

ORF ID^a^	Gene^b^	Product^b^	Log_2_FC^c^	FDR^d^	Absent in LbH^e^
Upregulated					
* MY479_RS15680*	—	DoxX family protein	2.72	6.05E‐11	—
* MY479_RS11795*	—	Putative quinol monooxygenase	1.31	1.26E‐06	Yes
Downregulated					
* MY479_RS03480*	*mauG1*	Cytochrome c peroxidase	−3.14	3.77E‐44	—
* MY479_RS03485*	*petE*	Methylamine utilisation protein	−1.87	2.83E‐14	—
* MY479_RS17595*	—	NADH ubiquinone oxidoreductase/hydrogenase 4 subunit G	−2.43	3.86E‐14	—
* MY479_RS17590*	—	NADH‐quinone oxidoreductase subunit D domain‐containing protein	−2.30	5.09E‐14	—
* MY479_RS17580*	—	Formate hydrogenase subunit E	−2.35	2.27E‐13	—
* MY479_RS03490*	—	Cytochrome c	−1.84	2.27E‐13	—
* MY479_RS04285*	*tpx*	Thiol peroxidase	−1.37	1.11E‐11	—
* MY479_RS04390*	*nuoD*	NADH‐quinone oxidoreductase subunit D	−1.28	1.23E‐11	—
* MY479_RS04395*	*nuoC*	NADH‐quinone oxidoreductase subunit C	−1.11	1.17E‐10	—
* MY479_RS17585*	—	NADH ubiquinone complex I subunit/Formate hydrogenase subunit F	−2.12	5.46E‐09	—
* MY479_RS17575*	—	NADH‐quinone oxidoreductase subunit H	−2.24	2.45E‐08	—
* MY479_RS17570*	—	Proton‐conducting transporter membrane subunit	−2.25	3.56E‐08	—
* MY479_RS09085*	*gshA*	Glutamate‐cysteine ligase	−1.62	8.68E‐08	—
* MY479_RS04385*	*nuoE*	NADH‐quinone oxidoreductase subunit NuoE	−1.11	4.22E‐07	—
* MY479_RS09090*	*gshAB*	Bifunctional glutamate—cysteine ligase GshA/glutathione synthetase GshB	−1.22	1.07E‐06	—
* MY479_RS04370*	*nuoJ*	NADH‐quinone oxidoreductase subunit J	−1.05	4.12E‐06	—
* MY479_RS09075*	*clpA*	ATP‐dependent Clp protease ATP‐binding subunit ClpA	−1.13	7.25E‐05	—
* MY479_RS09070*	*clpS*	ATP‐dependent Clp protease adapter ClpS	−1.17	1.37E‐04	—
* MY479_RS09080*	*ggt*	Gamma‐glutamyltransferase	−1.08	1.45E‐04	—
* MY479_RS17565*	—	PF13372 domain protein/alginate export family protein	−2.00	2.17E‐04	—
* MY479_RS09065*	—	GNAT family N‐acetyltransferase	−1.24	2.88E‐04	—
* MY479_RS13385*	—	NAD(P)/FAD‐dependent oxidoreductase	−1.48	1.38E‐03	Yes
* MY479_RS12375*	*perRB*	Peroxide‐responsive transcriptional repressor PerRB	−1.51	2.69E‐03	—
* MY479_RS14265*	*gst*	Glutathione S‐transferase family protein	−2.33	6.43E‐03	—

^a^Gene identifiers based on NCBI RefSeq annotation of *L. interrogans* sv. Hardjo strain KR40 (GCF_023158895.1).

^b^Description based on combined RefSeq and UniProt annotations.

^c^log_2_ fold change between in vivo and in vitro conditions.

^d^False discovery rate.

^e^Genes without orthologues in *L. borgpetersenii* sv. Hardjo.

Regarding the oxidative‐stress response, only a limited number of transcriptional shifts were observed. A minor reduction was detected for *perRB*, a regulator influencing oxidative‐stress survival through its effect on superoxide tolerance [43]. Several classical oxidative‐stress genes were downregulated, including *tpx*, a thiol peroxidase involved in peroxide detoxification, as well as the glutathione biosynthesis operon (*clpA*, *clpS*, *gshAB*, *gshA* and *ggt*). Among the DEGs were also several encoding DoxX family proteins, including *MY479_RS15680*, which showed the strongest upregulation within this group. DoxX family proteins are presumed to play a role in bacterial stress response based on studies in *Mycobacterium tuberculosis* [75].

At the level of respiratory complexes, marked reduction in expression was observed for the complex I operon (*nuoJ*, *nuoE*, *nuoD*, *nuoC*, *nuoH*, *nuoK* and *nuoF*). Additionally, a second NADH‐linked electron transfer operon (*MY479_RS17565-MY479_RS17595*) was also markedly downregulated. A similar repression has been reported upon serum exposure [30], whereas in the *perRAperRB* double mutant, these genes were upregulated [43], indicating that their expression is normally induced in response to oxidative stress.

The strongest downregulation was observed within a putative operon *MY479_RS03480-MY479_RS03495*, potentially implicated in methylamine utilisation (*mauG1*, *petE*). In other Gram‐negative such *mau*‐like operons support methylamine oxidation in environmental conditions where this compound is available, typically rich in decomposing organic matter [76].

#### 3.5.5. Differential Expression of Signal Transduction Genes

Since COG analysis revealed a pronounced decrease in the number of genes involved in signal transduction processes, a more detailed examination of these pathways was undertaken. The most prominent genes are summarised in Table 6, while the complete list is provided in Supporting Information 6: Table S6. Among the DEGs were several downregulated components of cyclic nucleotide‐dependent signalling, including cyclic diguanylate phosphodiesterases, diguanylate cyclases and adenylate/guanylate cyclases [77, 78]. In addition, numerous histidine kinases—the sensory components of two‐component systems [78, 79]—as well as STAS domain‐containing proteins were also downregulated.

**Table 6:** Selected differentially expressed signal transduction genes of *L. interrogans* sv. Hardjo after 24 h in the ovine DMC model.

ORF ID^a^	Gene^b^	Product^b^	Log_2_FC^c^	FDR^d^	Absent in LbH^e^
Upregulated					
* MY479_RS18145*	*mazE*	Transcriptional regulator/antitoxin MazE	1,25	5,96E‐04	Yes
* MY479_RS18150*	*mazF*	Endoribonuclease MazF	1,06	7,08E‐03	Yes
Downregulated					
* MY479_RS18470*	—	Cyclic diguanylate phosphodiesterase	−3.10	2.18E‐59	Yes
* MY479_RS19535*	—	STAS domain‐containing protein	−5.84	5.16E‐34	—
* MY479_RS18460*	—	STAS domain‐containing protein	−2.56	8.99E‐29	Yes
* MY479_RS18455*	—	Hypothetical protein	−2.84	2.71E‐25	—
* MY479_RS18450*	—	Tetratricopeptide repeat protein	−2.61	6.73E‐19	—
* MY479_RS18445*	*rsbU*	Serine phosphatase RsbU	−1.73	8.13E‐11	—
* MY479_RS12210*	—	Cyclic diguanylate phosphodiesterase	−2.08	1.56E‐08	—
* MY479_RS05570*	—	Cyclic diguanylate phosphodiesterase	−1.09	2.66E‐08	—
* MY479_RS12505*	—	Diguanylate cyclase	−1.89	4.21E‐08	Yes
* MY479_RS07660*	—	PAS domain S‐box protein	−1.30	8.74E‐08	Yes
* MY479_RS13855*	—	Adenylate/guanylate cyclase	−1.54	2.30E‐07	Yes
* MY479_RS19530*	—	Cyclic diguanylate phosphodiesterase	−1.34	6.14E‐07	—
* MY479_RS18465*	—	STAS domain‐containing protein	−5.34	1.07E‐06	Yes
* MY479_RS12220*	—	Response regulator	−2.30	2.79E‐06	—
* MY479_RS12225*	—	Histidine kinase	−2.03	2.79E‐06	Yes
* MY479_RS12215*	—	Histidine kinase	−2.13	3.96E‐06	—
* MY479_RS12515*	—	Diguanylate cyclase	−1.51	6.79E‐06	—
* MY479_RS12680*	*cyaA*	Adenylate/guanylate cyclase	−1.31	9.41E‐06	Yes
* MY479_RS19525*	—	Diguanylate cyclase	−1.23	2.74E‐05	—
* MY479_RS14270*	—	Histidine kinase	−1.62	1.12E‐04	—
* MY479_RS10645*	—	Histidine kinase	−1.99	3.53E‐04	—
* MY479_RS10660*	—	STAS domain‐containing protein	−1.89	2.55E‐02	—

^a^Gene identifiers based on NCBI RefSeq annotation of *L. interrogans* sv. Hardjo strain KR40 (GCF_023158895.1).

^b^Description based on combined RefSeq and UniProt annotations.

^c^log_2_ fold change between in vivo and in vitro conditions.

^d^False discovery rate.

^e^Genes without orthologues in *L. borgpetersenii* sv. Hardjo.

Several loci were downregulated at the operon level. *MY479_RS18445–MY479_RS18460*, previously annotated as *lb136–lb139* [80], has been implicated in the regulation of motility and chemotaxis under host conditions. Two adjacent genes, *MY479_RS18465* (STAS domain‐containing protein) and *MY479_RS18470* (a cyclic diguanylate phosphodiesterase), were among the most strongly downregulated in the entire dataset and lack orthologues in *L. borgpetersenii* sv. Hardjo. A second repressed locus, *MY479_RS12210–MY479_RS12225*, encodes encodes a signalling module that includes a c‐di‐GMP phosphodiesterase. Moreover, the putative operon *MY479_RS12505–MY479_RS12535*, which encodes diguanylate cyclases, exhibited consistent downregulation in four genes, three of which are missing orthologues from *L. borgpetersenii* sv. Hardjo.

Among the few upregulated genes was the putative *mazEF* operon encoding toxin–antitoxin (TA) system, which in other bacteria, plays an important role in persistence under unfavourable conditions, including growth arrest [81, 82]. In *L. borgpetersenii*, this locus is represented by a divergent orthologue sharing slightly above 30% sequence identity.

#### 3.5.6. Differential Expression of Chemotaxis and Motility Genes

Given the critical role of motility and chemotaxis in *Leptospira* pathogenesis, genes associated with these processes were examined in greater detail (Table 7), while the complete list of genes assigned to this category is provided in Supporting Information 6: Table S6. Overall, transcriptional changes in this group were less pronounced, with many genes not meeting the |log_2_FC| > 1 threshold and only rarely affecting entire operons. Most alterations reflected downregulation of motility‐associated genes, consistent with a moderate reduction in motility rather than its complete suppression.

**Table 7:** Selected differentially expressed chemotaxis and motility genes of *L. interrogans* sv. Hardjo after 24 h in the ovine DMC model.

ORF ID^a^	Gene^b^	Product^b^	Log2FC^c^	FDR^d^	Absent in LbH^e^
Upregulated					
* MY479_RS11245*	—	Methyl‐accepting chemotaxis protein	2.44	4.39E‐16	—
* MY479_RS00225*	—	Methyl‐accepting chemotaxis protein	1.04	3.06E‐04	—
* MY479_RS01260*	—	Flagellar motor switch protein FliG C‐terminal domain‐containing protein	1.19	1.45E‐02	—
Downregulated					
* MY479_RS03495*	—	Methyl‐accepting chemotaxis protein	−1.73	1.20E‐19	—
* MY479_RS17480*	—	Methyl‐accepting chemotaxis protein	−1.34	5.29E‐08	—
* MY479_RS07780*	*cheR*	Protein‐glutamate O‐methyltransferase CheR	−1.89	5.48E‐06	—
* MY479_RS03650*	*flbD*	Flagellar FlbD family protein	−1.00	6.85E‐05	—
* MY479_RS16360*	—	Chemotaxis protein	−1.44	1.58E‐03	—
* MY479_RS12305*	—	Flagellar Assembly Protein A N‐terminal region domain‐containing protein	−1.00	7.08E‐03	—
* MY479_RS10675*	*cheW3*	Chemotaxis protein CheW	−2.30	1.21E‐02	—
* MY479_RS10685*	*cheB*	Chemotaxis protein CheB	−1.18	1.82E‐02	—
* MY479_RS10665*	*cheA*	Chemotaxis protein CheA	−1.81	2.24E‐02	Yes
* MY479_RS10680*	*cheD*	Chemotaxis protein CheD	−1.93	2.27E‐02	—
* MY479_RS10655*	*cheY*	Chemotaxis protein CheY	−1.41	3.03E‐02	—

^a^Gene identifiers based on NCBI RefSeq annotation of *L. interrogans* sv. Hardjo strain KR40 (GCF_023158895.1).

^b^Description based on combined RefSeq and UniProt annotations.

^c^log_2_ fold change between in vivo and in vitro conditions.

^d^False discovery rate.

^e^Genes without orthologues in *L. borgpetersenii* sv. Hardjo.

With regard to flagellum‐associated processes, the majority of genes were slightly downregulated (Supporting Information 6: Table S6). Within the flagellar biosynthesis, *fliO* showed a decrease in expression. In the synthesis of basal body, *flgJ*, *flgI* and *flgA* were downregulated, while the hook assembly, *flgD* and *flgE* also exhibited decreased expression. The motor‐associated gene *fliG*, encoding a protein interacting with the MotA stator, was likewise downregulated. In addition, *flbD* and *motA* showed significant decreases in expression. In contrast, a *motB* paralog (*MY479_RS15755*), a *flaB* paralog (*MY479_RS15195*) and a *fliG* paralog (*MY479_RS01260*) displayed slight upregulation.

Genes involved in chemotaxis exhibited more pronounced transcriptional changes. Among all genes assigned to this functional category, only two were upregulated (*MY479_RS11245* and *MY479_RS00225*), both encoding methyl‐accepting chemotaxis proteins (MCPs). Conversely, two different MCP genes showed significant downregulation (*MY479_RS03495* and *MY479_RS17480*). In addition, several components of Che signalling cascade (*cheR*, *cheA*, *cheB*, *cheD*, *cheW* and *cheY*) exhibited coordinated downregulation.

#### 3.5.7. Differential Expression of Genes Involved in Energy and Metabolism

Given the pronounced transcriptional shifts observed in metabolic and energy‐related categories, further investigation was conducted on genes that may influence these processes (Table 3). The table summarises selected genes involved in lipid, carbohydrate and amino acid metabolism, as well as genes participating directly in cellular energy generation, while the complete list is provided in Supporting Information 6: Table S6.

With regard to energy production, the FoF_1_‐ATP synthase operon showed consistent upregulation, particularly in genes encoding core subunits (*atpH*, *atpF*, *atpE and atpB*). Given that pathogenic *Leptospira* primarily relies on fatty acids as its main source of carbon, deriving most of its energy through β‐oxidation pathways [83], we next examined genes involved in lipid metabolism. Several genes associated with fatty acid degradation, including *caiA* (acyl‐CoA dehydrogenases) and *acs* (acetate‐CoA ligase), were downregulated.

A more heterogeneous pattern was observed among lipid biosynthesis genes. *fabH* and *sch*, encoding ketoacyl‐ACP synthase III and β‐ketoacyl synthase, respectively, were upregulated. Both enzymes participate in the elongation of fatty acid chains. In contrast, *fabG*, responsible for the subsequent reduction steps in the fatty acid synthesis pathway, was slightly downregulated.

Pathogenic leptospires do not utilise hexose sugars as an energy source [84], although genes of the lower glycolytic pathway are retained. The pyruvate dehydrogenase (PDH) E1 complex (*MY479_RS08630-MY479_RS08635*) and *lpdA*, encoding a dihydrolipoyl dehydrogenase, were downregulated. Genes involved in glycerol‐3‐phosphate (G3P) metabolism also showed differential expression: the G3P antiporter *uphC* was upregulated, whereas *glpA2* and *gpsA*, encoding glycerol‐3‐phosphate dehydrogenases, were downregulated. In addition, increased expression was observed for the gluconeogenesis‐related genes *fbp* (class I fructose‐bisphosphatase) and *rpiB* (ribose‐5‐phosphate isomerase B).

Several DEGs were associated with amino acid metabolism. Genes involved in the synthesis of aromatic amino acids, *trpA*, *trpC*, *trpD*, *trpB*, *aroE* and *aroK* (Supporting Information 6: Table S6) were downregulated. Genes involved in lysine (*dapF*) and cysteine (*cysE*) biosynthesis, as well as branched‐chain amino acid catabolism (*ivd*), also showed reduced expression. In contrast, *ilvB* (large subunit of biosynthetic acetolactate synthase) and *glnA* (glutamine synthetase) were upregulated. Within the histidine biosynthetic pathway, *hisD* and *hisG* were upregulated, whereas *hisI* and *hisC* were downregulated.

Part of the metabolic changes was associated with cell envelope remodelling. Within the *rfb* locus (*MY479_RS07120-MY479_RS07500*) involved in polysaccharide O‐antigen LPS biosynthesis [85], several genes showed slight upregulation (Supporting Information 6: Table S6). Additional carbohydrate‐related genes outside the *rfb* locus were also differentially expressed, including several glycosyltransferases and a strongly downregulated nucleoside‐diphosphate sugar epimerase (*MY479_RS15970*). Genes involved in peptidoglycan metabolism, including *alr* (alanine racemase), were also upregulated [86].

Finally, the carbonic anhydrase gene *sul1* was strongly upregulated. Although not directly linked to any single metabolic pathway described above, carbonic anhydrase contributes more broadly to central carbon metabolism by regulating CO_2_/HCO_3_^−^ balance and supplying bicarbonate for carboxylation‐dependent biosynthetic reactions.

#### 3.5.8. Validation of RNA‐Seq Findings Under Controlled In Vitro Stress Conditions

To validate the transcriptional patterns observed in the RNA‐seq analysis, we next assessed the expression of selected stress‐associated and surface‐exposed genes by RT‐qPCR under defined in vitro conditions. Significant changes in gene expression were observed across all tested loci, with strain‐ and condition‐specific patterns confirmed by one‐way ANOVA followed by post hoc testing (*p* < 0.05; Figure 6).

Figure 6Expression of selected oxidative stress‐responsive (A) and iron limitation‐responsive (B) genes after in vitro exposure. Oxidative stress was induced with H_2_O_2_ at different concentrations and exposure times and iron limitation with 2,2^′^‐dipyridyl. Gene expression was quantified by RT‐qPCR and normalised to strain‐specific untreated controls. Data represent mean ± SD from three biological replicates. Asterisks indicate significant differences ( ^∗^ *p* < 0.05,  ^∗∗^ *p* < 0.01,  ^∗∗∗^ *p* < 0.001).

**Figure:**
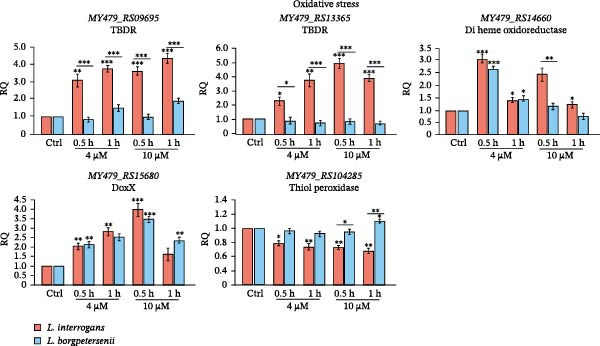
(A)

**Figure:**
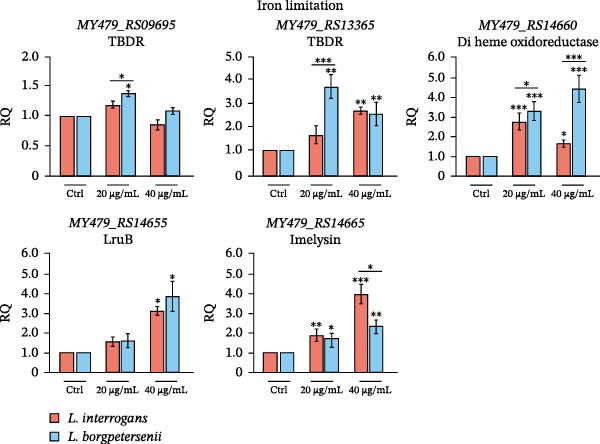
(B)

Under oxidative stress (H_2_O_2_ treatment) *L. interrogans* sv. Hardjo exhibited strong upregulation of both TBDRs (*MY479_RS09695*, *MY479_RS13365*), di‐haem oxidoreductase (*MY479_RS14660*) and the DoxX‐family protein (*MY479_RS15680*) genes, whereas in *L. borgpetersenii* no statistically significant changes were observed (Figure 6A). In contrast, the expression of *tpx* (*MY479_RS04285*) decreased significantly in *L. interrogans* sv. Hardjo, while remaining stable or slightly increased in *L. borgpetersenii* sv. Hardjo.

In iron‐limiting conditions (40 µg/mL dipyridyl) both species showed robust induction of TBDR (*MY479_RS13365*) and di‐haem oxidoreductase genes, with *L. borgpetersenii* sv. Hardjo displaying a higher fold change in the di‐haem oxidoreductase (Figure 6B). The imelysin (*MY479_RS14665*) and LruB (*MY479_RS14655*) genes were consistently among the most highly upregulated in both strains, aligning with RNA‐seq data, with a stronger induction observed at 40 µg/mL dipyridyl.

## 4. Discussion

Understanding the early events of *Leptospira* infection is essential for elucidating the mechanisms of pathogen survival and further colonisation of host tissues and organs. Genomic studies have revealed numerous non‐canonical, unique genes in pathogenic strains [1, 14, 87–89], but their role in response to the host environment cannot be resolved by sequence data or by mimicking single host signals in vitro, highlighting the need for in vivo‐like models. In this study, we used a DMC model in sheep, which provided a closer approximation of the host milieu, exposing bacteria to multiple factors, including altered temperature, osmotic pressure, and a complex mixture of soluble host‐derived factors diffusing through a semi‐permeable membrane. Notably, under these conditions, only *L. interrogans* sv. Hardjo survived and remained motile after 24 h, while *L. borgpetersenii* sv. Hardjo was rapidly eliminated (Figure 1), consistent with previous DMC studies in rats [37]. Thus, even in a natural reservoir host such as sheep, *L. borgpetersenii* fails to withstand the early peritoneal environment, pointing to fundamental species‐level differences in early survival strategy.

In our experiment, we used two distinct strains of *L. interrogans* sv. Hardjo: KR40 and N116 [18], and merged their datasets for downstream analysis. This approach was intended to capture transcriptional changes that are universal and not driven by strain‐specific differences. As illustrated by the PCA analysis (Figure 3A) and supported by the heatmap visualisation (Figure 3B), the data show that the transcriptional shift induced by the host environment is the dominant source of variation between samples. Partial intermixing of KR40 and N116 after 24 h in vivo suggests that, at least during the early stage of infection, both strains exhibit a similar response pattern. This overall pattern was reflected at the level of differential gene expression: 1066 genes were significantly altered under host conditions (FDR < 0.05; Figure 4). To focus on genes showing the most biologically meaningful transcriptional shifts, an absolute log_2_FC threshold of ≥1 was applied. This filtering reduced the dataset to 397 genes, comprising 168 upregulated and 229 downregulated transcripts, of which nearly 40% had no orthologues the *L. borgpetersenii* sv. Hardjo genome (Supporting Information 5: File S5), indicating that a substantial portion of the early in vivo response involves functional elements unique to *L. interrogans* sv. Hardjo. Previous genomic studies investigating the diversity and evolutionary history of *L. interrogans* sv. Hardjo have characterised gene content and genomic organisation within this lineage [16–18]. Our results provide functional context for these genomic observations, showing that a considerable fraction of genes activated during early host exposure belong to loci without orthologues *in L. borgpetersenii* sv. Hardjo. It should be noted; however, that the genomic repertoire of *L. interrogans* and *L. borgpetersenii* may vary depending on strain origin and geographical background, and therefore the extent of orthologue absence or presence observed here may not fully represent the diversity of these species.

The initial COG‐based analysis (Figure 5) provided a first indication that early host‐associated response of *L. interrogans* sv. Hardjo is characterised by a redirection of regulatory and metabolic priorities towards structural and biosynthetic adjustment, accompanied by a reallocation of energy resources and a downshift in motility‐related functions. Accordingly, these categories were selected for more detailed examination, as their prominence suggests they may represent a crucial adaptive strategy during the initial phase of host infection following pathogen entry.

Bacterial adaptation in early infection involves not only internal transcriptional regulation, but also the ability to respond to host‐derived signals, including innate immune pressures. During infection, *Leptospira* encounters multiple layers of host defence, including physical barriers, biochemical factors and soluble components such as complement proteins and AMPs [90, 91]. Both complement and AMPs can directly contribute to bacterial killing: the complement cascade is one of the earliest innate immune mechanisms, while AMPs are small cationic host‐defence molecules that act mainly by disrupting microbial membranes [92–94]. These peptides exert their antimicrobial effect primarily through electrostatic interactions with negatively charged bacterial membranes, leading to membrane destabilisation and leakage of cellular contents. Although the DMC model does not allow the passage of host cells or diffusion of large immune components such as complement proteins, owing to the limited pore size of the membrane, the detection of host defensins in the dialysate indicates that small AMPs can diffuse across this barrier, representing the major innate immune pressure in this environment (Supporting Information 3: Figure S3). This effect was especially evident for *L. borgpetersenii* sv. Hardjo, whose dialysate showed a stronger signal associated with the presence of α‐ and β‐defensins. Importantly, our in vitro assays demonstrated that abiotic stressors typical of the dialysate, including osmotic or oxidative conditions (Figure 2B,C), did not account for the distinct survival patterns observed between *L. interrogans* sv. Hardjo and *L. borgpetersenii* sv. Hardjo. This suggests that additional host‐derived factors, such as AMPs, may play an important role in shaping their differential responses. Previous studies have shown that pathogenic *Leptospira* spp. evade such innate immune challenges through proteolytic mechanisms targeting host AMPs [95–98]. Among their virulence factors, secreted proteases and particularly metalloproteases play a central role in neutralising host defence molecules and promoting bacterial survival [98–100]. Genomic and biochemical studies have identified at least 12 putative metalloproteases in *L. interrogans*, several with elastinolytic and caseinolytic activity indicative of broad substrate specificity [99]. For example, *L. interrogans* produces metalloproteases capable of cleaving the human cathelicidin LL‐37, a cationic AMP that represents the only cathelicidin in humans but has functional homologues across several mammalian species, including cattle, pigs and sheep [98]. Like defensins, LL‐37 disrupts bacterial membranes through electrostatic interactions. In this context, our transcriptomic analysis identified several upregulated genes encoding predicted metalloproteases and metallopeptidases in *L. interrogans* sv. Hardjo recovered from the ovine DMC, including *MY479_RS02605* (M48 family metallopeptidase), *MY479_RS13270* (M14 family zinc carboxypeptidase), *MY479_RS17645* (M43 family zinc metalloprotease), *MY479_RS16225* (M15 family metallopeptidase), *MY479_RS04050* (trypsin‐like peptidase) and *MY479_RS06895* (S41 family peptidase) (Supporting Information 5: File S5). Although the specific substrates and biological functions of these enzymes remain uncharacterised, their induction under DMC conditions suggests that proteolytic pathways are transcriptionally responsive during exposure to host‐derived antimicrobial pressures. Similar protease‐mediated responses have been reported in other bacterial pathogens, where metalloproteases can contribute to survival under AMP stress [101]. Nevertheless, these observations do not establish any mechanistic link to AMP neutralisation, and functional studies will be required to determine whether, and under what circumstances, these proteases contribute to leptospiral interactions with host antimicrobial molecules.

Among the most strongly induced genes in the ovine DMC environment were *ligA* and *ligB* followed by *lenC* and *lenF*, all of which encode immunoglobulin‐like surface adhesins central to *Leptospira* pathogenesis (Table 2). Unlike the complement‐binding paralogs LenA and LenB, LenC and LenF belong to the double‐Len‐motif subgroup with the highest affinities for laminin and fibronectin [60, 88]. Their expression is normally undetectable in standard culture conditions but becomes strongly induced under host‐like, particularly iron‐limited environments [42], consistent with a nutrient‐responsive adhesion phenotype. The simultaneous activation of *ligA/B* and *lenC/F;* therefore, reflects a coordinated remodelling of the leptospiral surface in a response to host cues, integrating ECM targeting with response to innate‐immune and nutritional pressures. These adhesins bind multiple extracellular‐matrix components, including fibronectin, laminin and collagen and LigA/B can also recruit complement regulators such as C4BP and factor H [41, 102]. Their induction under ovine DMC conditions parallels observations from rat DMC models [33], underscoring the conserved nature of early adhesion and immune‐evasion programmes in *L. interrogans* under host‐derived conditions. Conversely, *lipL32*, encoding a major outer membrane lipoprotein, was significantly downregulated in the ovine DMC environment. This trend mirrors earlier findings showing that *lipL32* expression decreases upon exposure to host serum [30], during contact with macrophages [103] and also in vivo [104], where *lipL32* repression marked the transition from an environmental to a host‐adapted state. Together with the strong induction of *lenC* and *lenF*, this pattern supports selective remodelling of the outer membrane, in which highly immunogenic but dispensable lipoproteins are suppressed while host‐inducible adhesins are expressed. In this context, *MY479_RS02610*, encoding a predicted LipL46‐family lipoprotein, was also upregulated. Homologues of this protein in *L. interrogans* bind plasminogen [105], suggesting a possible contribution to host‐protein interactions.

In addition to the canonical Lig and Len adhesins, the ovine DMC model revealed strong induction of lsa21 and four genes with homology to lipL53 known to bind laminin, fibronectin and collagen IV [106, 107], as well as MY479_RS13150 (DUF1565‐domain lipoprotein gene; Table 2), previously shown to bind collagen IV, laminin and plasminogen [63]. Two additional induced loci complement this pattern: mce, encoding the Mce protein, a proven virulence factor that binds extracellular matrix components and promotes mammalian‐cell infection [61, 108], and glnA encoding glutamine synthetase (Table 3), which was reported as a plasminogen‐binding protein [109].

Together, these changes indicate that *L. interrogans* sv. Hardjo integrates surface remodelling with metabolic adjustment during early host exposure. Notably, the downregulation of selected T2SS components (Supporting Information 6: Table S6) together with the upregulation of proteins involved in membrane insertion (such as *yidC*, *yidD* and *lolA*) suggests that, during the early in vivo phase, *L. interrogans* sv. Hardjo dynamically adjusts its secretion machinery, with distinct sets of lipoproteins potentially targeted for insertion through alternative pathways. The identification of *L. interrogans*‐specific adhesins highlights the expanded surface repertoire characteristic of pathogenic *Leptospira*. Comparative genomic analyses show that *L. borgpetersenii* lacks or carries truncated orthologues for many of these genes [12, 64]. This reduced repertoire, together with its more limited regulatory capacity, may restrict its ability to modulate surface structures in response to host‐derived stimuli present in the DMC environment. Indeed, *L. borgpetersenii* exhibits minimal induction of *lig* genes even under physiological temperature or serum exposure [110].

Iron limitation represents the next key selective pressure during the early phase of infection. The host actively restricts iron availability by sequestering it intracellularly within erythrocytes or by binding it to transport and storage proteins, thereby minimising the pool accessible to invading pathogens [111]. Both in vitro and in vivo studies have demonstrated that leptospirosis disrupts host iron homeostasis and confirmed that iron is essential for the growth and survival of *Leptospira* spp [42, 68, 112, 113]. Our in vitro assay further showed that *L. interrogans* sv. Hardjo displays higher survival under iron‐limiting conditions than *L. borgpetersenii* sv. Hardjo (Figure 2A), which aligns with the differential expression of several genes implicated in iron‐related processes, as detected in our dataset (Table 4; Supporting Information 6: Table S6). Consistent with an increased need for iron acquisition, we observed downregulation of four FecR domain‐containing genes. FecR‐like regulators in *Leptospira* are thought to form an analogue of the FecI‐FecR system described in *E. coli* [74], where they modulate σ‐factor activity in response to environmental cues. Under stress conditions—particularly iron or nutrient limitation—such systems are likely to regulate genes essential for survival within the host environment. Although the specific downstream targets of identified FecR‐like regulators remain unknown, our dataset revealed strong upregulation of several TBDR genes. TBDRs are known, in addition to its proteolytic activity, to mediate the uptake of various substrates, including haem, xenosiderophores and vitamin B12 [72], suggesting that these receptors may fulfil additional roles during the initial host‐triggered bacterial response. Their relevance is further supported by transcriptomic studies showing their upregulation in the DMC model [33], serum exposure assay [30], and experiment conducted under iron‐limiting conditions [42]. In *L. interrogans* sv. Hardjo, 11 distinct TBDRs were identified, although only four—*MY479_RS09695*, *MY479_RS11530* (no orthologue in *L. borgpetersenii* sv. Hardjo), *MY479_RS14650* and *MY479_RS13365*—showed significant transcriptional regulation. Transport through these receptors requires enewrgy transduction from the TonB–ExbB–ExbD complex [72]; however, we observed only a slight upregulation of *exbD* and a slight downregulation of *MY479_RS19590* (ExbB family protein; Supporting Information 5: File S5), suggesting that *L. interrogans* sv. Hardjo modulates primarily receptor abundance rather than the core energy‐transducing machinery in response to host‐derived conditions.

In addition, we observed increased expression of an imelysin‐like containing putative operon *MY479_RS14655–MY479_RS14665*, which responds strongly to iron limitation [42] and is located in close proximity to an upregulated gene encoding one of the TBDR (*MY479_RS14650*). Among the encoded proteins, particular attention should be given to imelysin LruB. Mutants lacking this gene exhibit a loss of *in vitro* viability [114]. Beyond its iron‐related role [42], LruB has been shown to interact with laminin, fibronectin, collagen types I and IV [115]. Together with LruC (*MY479_RS15895*, upregulated), these proteins have been detected in the ocular fluids of horses with equine recurrent uveitis, where they elicit strong antibody responses [70, 71].

To investigate whether these genes may contribute to the superior survival of *L. interrogans* sv. Hardjo, we conducted PCR expression assays (Figure 6). Interestingly, under‐iron‐limiting conditions, *L. borgpetersenii* sv. Hardjo displayed stronger upregulation of TBDRs, di‐haem oxidoreductase, and LruB genes. However, in response to hydrogen peroxide, *L. borgpetersenii* sv. Hardjo did not coordinate iron‐related genes with oxidative stress responses as effectively as *L. interrogans* sv. Hardjo. This suggests that *L. borgpetersenii* sv. Hardjo may lack integrated iron‐oxidative stress regulation, potentially limiting its ability to withstand simultaneous host‐derived pressures. Iron‐dependent systems in pathogenic *Leptospira* have been shown to overlap with responses to diverse environmental stressors rather than functioning as isolated pathways [29, 30, 69].

*L. borgpetersenii* is characterised by marked genomic reduction and loss of environmental survival capacity [12]. Given that pathogenic leptospires can transiently survive within macrophages during early infection as part of their strategy to evade innate immune defences [116], it is plausible that *L. borgpetersenii* may rely more heavily on such intracellular niches. These environments are relatively iron‐rich but simultaneously oxidatively challenging, which could further explain the observed uncoupling. In our ovine DMC model the oxidative‐stress response appeared to play only a minor role, as only a limited subset of classical oxidative‐stress genes showed repression‐most prominently *tpx* and the glutathione‐biosynthesis operon (Table 5). Moreover, intracellular environment is not dominated by AMPs, reducing the selective pressure associated with AMP‐mediated surface damage.

Upon entering the host organism, bacteria must rapidly adjust their behaviour to dynamic environmental conditions, including fluctuations in the concentration of various chemical compound [117]. Such adjustments typically involve remodelling of signal transduction and chemotaxis systems, which are essential for navigating the host environment. A large number of DEGs were associated with modifications of signal transduction pathways, the majority of which showed downregulation (Table 6). Several diguanylate cyclases were decreased, suggesting a reduction in the synthesis of c‐di‐GMP. In contrast, we also identified downregulation of several phosphodiesterases responsible for c‐di‐GMP degradation. In bacteria, including *Leptospira*, high c‐di‐GMP levels typically promote biofilm formation, surface adhesion and reduced motility, whereas low levels favour a motile state [118–120]. The simultaneous decrease of both classes of enzymes generally indicates that the objective is neither a strong increase nor a strong reduction of intracellular c‐di‐GMP, but rather its stabilisation at an intermediate level, limiting the dynamics of this signalling pathway. Such a pattern may reflect an early host‐associated state in which the bacterium refrains from committing to either active biofilm formation or full motility activation, instead maintaining a ‘silent,’ energy‐efficient profile that supports evasion of early immune detection. Concurrent downregulation of several adenylate/guanylate cyclases in our dataset, which would limit the production of cAMP, another signalling nucleotide, further supports this interpretation. Since cyclic dinucleotides can stimulate host innate immune responses [121] a broader reduction in nucleotide‐based signalling may help minimise early immune recognition.

Within this category, the entire set of genes in the *MY479_RS18445–MY479_RS18460* locus (corresponding to *lb139–lb136*), which encodes a putative operon previously implicated in the regulation of motility and chemotaxis [80], was downregulated. Mutations in *lb139*, which encodes a sensor protein containing a phosphatase domain, were shown to result in the downregulation of multiple motility‐ and chemotaxis‐related genes, supporting its regulatory role. In our transcriptomic dataset, a distinct set of genes showing pronounced differential expression associated with chemotaxis and motility processes was downregulated (Table 7, Supporting Information 6: Table S6) such as components of the chemotaxis signalling cascade *cheA*, *cheB*, *cheD*, *cheW*, *cheY* or MCP genes—exhibited marked downregulation. Chemotactic behaviour relies on an integrated phosphorelay system in which membrane‐associated MCPs, the most common chemoreceptors in prokaryotes, sense environmental cues and transmit the signal into the cytoplasm. CheW links MCP to the sensory kinase CheA, and CheA further transmits the signal to CheB or CheY. Phosphorylated CheY then modulates with the flagellar switch to control the direction of rotation, while CheB acts as a methylesterase that removes methyl groups from MCPs, thereby adjusting their sensitivity, while CheR adds methyl groups. CheD converts glutamine residues in the receptor, making it more prone to methylation [80, 122]. In one study [123], inactivation of the first gene histidine kinase resulted in suppressed transcription of all downstream genes within *la2421–la2429* operon (corresponding to *MY479_RS10645-MY479_RS10685*). *Leptospira* mutant showed a marked reduction in reversal frequency while maintaining normal swimming speed and trajectory. Importantly, the mutant remained fully virulent, indicating that this locus is not strictly required for infection.

Changes were also mirrored at the level of the flagellar apparatus. We observed a modest but statistically significant downregulation of several genes involved in flagellar regulation and assembly (*flbD*, *fliG*, *fliD*, *flgE*, *flgN*, *flgJ*, *fliE*, *flgI*, *fliO* and *motA*) (Table 7, Supporting Information 6: Table S6). Although previous DMC model transcriptomic studies have reported upregulation of motility‐related genes during host incubation [33], we did not observe a similar trend under our experimental conditions. This discrepancy may reflect differences in the *in vivo* phase, as in the cited study leptospires were recovered after 9–10 days of host incubation, whereas our data likely represent an earlier stage when bacteria may transiently deprioritises gradient sensing and directional control, potentially reallocating energy towards structural and metabolic adjustments rather than active navigation within the host environment, accompanied by a general reduction in active motility. Consistent with this assumption, a transcriptomic study examining *L. interrogans* during its initial interaction with macrophages also reported downregulation of motility‐associated genes [103].

In addition to the hypothesis that these patterns reflect energy reallocation, an alternative explanation is that the observed downregulation of chemotaxis‐ and motility‐associated genes may also serve to minimise early immune detection. Previous studies have shown that *Leptospira* spp. can evade the host immune response, at least in part, by failing to effectively activate TLR5 [124], which in other bacteria is responsible for flagellin recognition. One of the protective mechanisms against such recognition in *Leptospira* is the periplasmic localisation of flagella, which prevents exposure of FlaB subunits to host receptors. However, when the bacteria are degraded by AMPs such as LL37 or the bovine‐derived Bmap28, TLR5 signalling can be triggered in both human and bovine cells. It is therefore plausible to hypothesise that during the early phase of infection, *Leptospira* may initially refrain increasing chemotaxis and motility‐related gene expression, as observed for *flaB* [124], to minimise immune detection. *L. interrogans* encodes five *flaB* paralogs and interestingly, we detected only slight upregulation of one of these genes (*MY479_RS15195*,Supporting Information 6: Table S6), corresponding to *lic12947*. In the earlier DMC‐membrane study [33], this particular paralog was reported to be expressed at markedly lower levels than the remaining four *flaB* copies, suggesting that it may have a distinct, specifically regulated role during early in vivo phase.

Early interaction with the host also requires *L. interrogans* to remodel its metabolism to match the environmental conditions it encounters. Unlike many bacteria, *Leptospira* does not rely on C6 carbohydrates as an energy source. Instead, ATP production in this genus depends primarily on fatty‐acid catabolism, while the lower glycolytic pathway remains active [83, 125]. In our model, we observed a marked reduction of several components of these energy‐generating pathways, including β‐oxidation, pyruvate oxidation and glycerol‐3‐phosphate utilisation (Table 3), whereas the genes associated with the TCA cycle remained largely unchanged.

Both β‐oxidation and the TCA cycle generate NADH, which normally supplies electrons to complex I of the respiratory chain. In our dataset, complex I was predominantly downregulated (Table 5), while complexes II, III and IV showed no substantial changes in expression. Similar alterations in different levels of TCA cycle or oxidative phosphorylation have also been reported following exposure of *Leptospira* to serum [30]—likely reflecting the iron‐limited nature of this environment—and after interaction with macrophages [103], where reduced expression was interpreted as a consequence of the bacterium lowering its O_2_ requirement upon contact with host cells. Nevertheless, energy production appears to be sustained, as indicated by the clear upregulation of the locus encoding the FoF_1_‐ATP synthase complex (Table 3). The mechanism by which *L. interrogans* sv. Hardjo compensates for reduced NADH‐driven respiration remains unclear, as we did not identify any clear upregulation patterns in alternative routes supplying electrons to oxidative phosphorylation.

In parallel, some genes involved in elongation of fatty acid chains and gluconeogenesis biosynthesis were upregulated. Reflecting this redirected carbon flux, we also observed moderate upregulation across the *rfb* locus (Table 3, Supporting Information 6: Table S6), responsible for O‐antigen LPS biosynthesis [85], suggesting increased demand for NDP‐sugar substrates. By contrast, genes acting at the early steps of lipid A assembly and export (*lpxA*, *lpxK* and *lptB*) showed slight downregulation, whereas *lmtA*, involved in lipid A modification, was upregulated, suggesting that *Leptospira* prioritises remodelling rather than de novo synthesis of lipid A during the early phase following host entry. Taking this into account, together with the substantial number of upregulated lipoproteins, it appears likely that *L. interrogans* sv. Hardjo redirects carbon and lipid resources towards membrane and surface remodelling at this stage.

**Figure 7:**
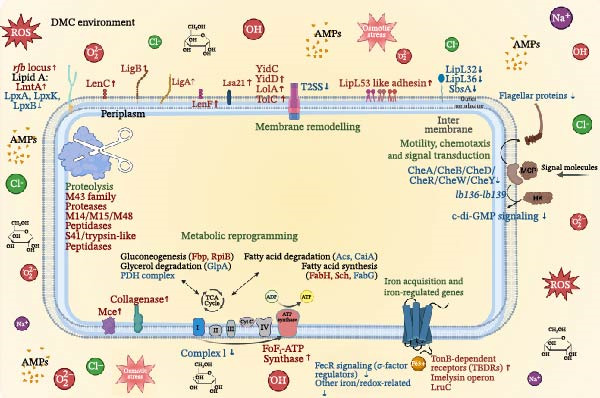
Major transcriptional and functional changes during the early host‐associated response of *Leptospira interrogans* sv. Hardjo. The scheme summarises dominant transcriptional changes during growth in the dialysis membrane chamber (DMC), a host‐like environment containing antimicrobial peptides (AMPs), reactive oxygen species (ROS), osmotic stress and iron limitation. Early host‐associated responses involve intensive membrane remodelling, with suppression of the type II secretion system (T2SS) and upregulation of nonclassical membrane transport and insertion factors, as well as induction of host‐binding lipoproteins (LigA/B, LenC/F, Lsa21 and LipL53‐like) or *rfb* locus. Transcriptional increases also extend to proteases/peptidases and TonB‐dependent receptors (TBDRs). Iron‐responsive imelysin genes are upregulated, whereas chemotaxis and motility systems are suppressed, including the methyl‐accepting chemotaxis protein (MCP), histidine kinases (HK) and the CheA–CheW–CheY cascade. Cyclic‐di‐GMP (c‐di‐GMP) and respiratory Complex I are reduced, while FoF_1_‐ATP synthase is increased, indicating metabolic reprogramming under host‐derived pressures. Together, these changes illustrate rapid adjustment to early host pressures (*Created with BioRender. Siemińska*, *I. [2025*]).

Our results show that, during the initial in vivo stage, *L. interrogans* sv. Hardjo activates a coordinated set of transcriptional responses that allow it to adjust to the complex mixture of pressures present in the ovine peritoneal environment (Figure 7). In contrast, *L. borgpetersenii* sv. Hardjo appeared unable to mount comparable responses, and the absence of key adaptive mechanisms likely contributed to its failure to survive in this model, even over a short incubation period and within a host species considered its natural reservoir. The DMC model, by exposing bacteria to simultaneous cues related to nutrient restriction, osmotic changes and small host‐derived immune factors such as AMPs, captured early responses that are not detectable in standard in vitro assays. A central feature of this response is extensive surface remodelling. The strong induction of host‐inducible adhesins, highlights a rapid reconfiguration of the leptospiral outer membrane towards enhanced ECM binding, immune evasion and nutrient acquisition. The selective induction of multiple proteases and metallopeptidases, alongside evidence of defensin diffusion into the DMC, suggests that proteolytic defences may be part of an early response to AMPs stress, although functional validation is required. Beyond surface changes, *L. interrogans* sv. Hardjo modulates nutrient‐acquisition systems, particularly those involved in iron uptake, and restructures signalling pathways by broadly reducing chemotaxis‐ and motility‐associated transcription. These adjustments appear to prioritise energy conservation and minimise immune detection during the earliest phase of infection.

## 5. Conclusion

Overall, our findings show that early survival of *L. interrogans* sv. Hardjo depends on a tightly coordinated transcriptional reprogramming that integrates surface remodelling, nutrient acquisition and energy allocation in response to complex host‐derived pressures. Using an ovine DMC model, we demonstrate that *L. interrogans*, but not *L. borgpetersenii*, rapidly activates adaptive programmes that support early survival in the host environment. By contrast, the absence of analogous adaptive signatures in *L. borgpetersenii* suggests that this species may rely on a fundamentally different survival strategy, possibly one that favours rapid transition into intracellular niches rather than temporary extracellular persistence.

## Funding

This work was supported by Grant OPUS 17 2019/33/B/NZ9/02159 from the National Science Centre, Poland.

## Ethics Statement

All animal experiments were conducted in accordance with the International Guidelines for Biomedical Research Involving Animals and were approved by the 2nd Local Animal Care and Use Committee in Krakow, Poland (Resolution Number 22/2021).

## Conflicts of Interest

The authors declare no conflicts of interest.

## Supporting Information

Additional supporting information can be found online in the Supporting Information section.

## Supporting information


**Supporting Information 1** Table S1: Sequences of forward and reverse primers of selected genes of *L. interrogans* sv. Hardjo.

**Supporting Information 2** Table S2: Analysis of bacterial count and key viability parameters in *L. interrogans* sv. Hardjo and L. borgpetersenii sv. Hardjo.

**Supporting Information 3** Figure S3: Dot blot detection of host α‐ and β‐defensins in supernatants recovered from DMCs after in vivo incubation in the peritoneal cavity of sheep. Positive control (PC) – purified defensin standard; C‐M – control medium (fresh, non‐incubated culture medium); DMC‐M – incubation medium (medium incubated in DMCs for 24 h without bacteria).

**Supporting Information 4** Table S4: Statistics on sequencing reads and genome alignment in *L. interrogans* sv. Hardjo.

**Supporting Information 5** File S5: All differentially expressed genes (DEGs) in *L. interrogans* sv. Hardjo. The file lists all statistically significant DEGs (FDR < 0.05), sorted in ascending order of FDR values, in two sheets for upregulated and downregulated genes. ^ᵃ^Gene identifiers based on NCBI RefSeq annotation of *L. interrogans* sv. Hardjo strain KR40 (GCF_023158895.1); ^ᵇ^Description based on combined RefSeq and UniProt annotations; ^ᶜ^log₂ fold change between in vivo and in vitro conditions; ^ᵈ^False discovery rate; ^e^COG category legend: A – RNA processing and modification; C – Energy production and conversion; D – Cell cycle control and division; E – Amino acid transport and metabolism; F – Nucleotide transport and metabolism; G – Carbohydrate transport and metabolism; H – Coenzyme transport and metabolism; I – Lipid transport and metabolism; J – Translation, ribosomal structure and biogenesis; K – Transcription; L – Replication, recombination and repair; M – Cell wall/membrane/envelope biogenesis; N – Cell motility; O – Posttranslational modification, protein turnover and chaperones; P – Inorganic ion transport and metabolism; Q – Secondary metabolites biosynthesis, transport and catabolism; R – General function prediction only; S – Function unknown; T – Signal transduction mechanisms; U – Intracellular trafficking, secretion and vesicular transport; V – Defence mechanisms; ^f^Genes without orthologues in L. borgpetersenii sv. Hardjo (bolded).

**Supporting Information 6** Table S6: Complete list of differentially expressed genes (DEGs) in *L. interrogans* sv. organised according to the functional categories discussed in the Results section. ^ᵃ^Gene identifiers based on NCBI RefSeq annotation of *L. interrogans* sv. Hardjo strain KR40 (GCF_023158895.1); ^ᵇ^Protein product description based on combined RefSeq and UniProt annotations; ^ᶜ^log₂ fold change between in vivo and in vitro conditions; ^ᵈ^False discovery rate; ^ᵉ^Genes without orthologues in L. borgpetersenii sv. Hardjo

## Data Availability

The genome assemblies produced by the authors and used in this study are publicly available in the NCBI database under the following BioProject Accession Numbers: *Leptospira interrogans* sv. Hardjo strains KR40 (BioProject: PRJNA828004) and N116 (BioProject: PRJNA828002), and *Leptospira borgpetersenii* sv. Hardjo strains KR39 (BioProject: PRJNA828006) and 58K3 (BioProject: PRJNA828000). Filtered RNA‐seq reads are available in the Zenodo repository under DOI https://doi.org/10.5281/zenodo.17909699.
