# Supplementary material for: Divergent Fates of Hardjo Leptospires: Early Transcriptomic Response of Leptospira interrogans in an Ovine Dialysis Membrane Chamber Model
Source: Transbound Emerg Dis. 2026 Apr 9;2026:2998023. doi: 10.1155/tbed/2998023 (PMC13066512; doi:10.1155/tbed/2998023)
Supplement: Supplementary file 1 — Supporting Information 1 Table S1: Sequences of forward and reverse primers of selected genes of L. interrogans sv. Hardjo. [file TBED-2026-2998023-s001.docx]

| **Genes** | **Primers (5´-3´)** | **Product size (bp)** |
| --- | --- | --- |
| *MY479_RS14660* | 5´- TATAAGGCCCGTTCCCCAGA -3´  5´- GGACCCGATCTTGCAGACAA -3´ | 87 |
| *MY479_RS14655* | 5´- TGTCCACCAAAAGTTCCGGT -3´  5´- GACGGTGCCAGATATTCGGA -3´ | 131 |
| *MY479_RS14665* | 5´- CTGAAACCGTGAGTCCTGCT -3´  5´- TCCGGTCAAGGTTCTTTCGG -3´ | 124 |
| *MY479_RS09695* | 5´- TACAGTCGCTTGAGAACCCC -3´  5´- TTCAGTGACCCTTCCGTTGG -3´ | 123 |
| *MY479_RS13365* | 5´- TTTACCGGTTCCTTCGTGCA-3´  5´- TTACGGACGCGGTTTTAGGG -3´ | 73 |
| *MY479_RS04285* | 5´- AACTAGGGTTGTTACGCCGG -3´  5´- ATACTTCCGTCTGTGCCCTC -3´ | 77 |
| *MY479_RS15680* | 5´- TTTTCCTCCGCCTTTGACCA -3´  5´- TGGTTTGGAAATCAGCAGGGA -3´ | 90 |
