# Supplementary material for: Divergent Fates of Hardjo Leptospires: Early Transcriptomic Response of Leptospira interrogans in an Ovine Dialysis Membrane Chamber Model
Source: Transbound Emerg Dis. 2026 Apr 9;2026:2998023. doi: 10.1155/tbed/2998023 (PMC13066512; doi:10.1155/tbed/2998023)
Supplement: Supplementary file 2 — Supporting Information 2 Table S2: Analysis of bacterial count and key viability parameters in L. interrogans sv. Hardjo and L. borgpetersenii sv. Hardjo. [file TBED-2026-2998023-s002.docx]

| **Strain** | **Period of** **incubation** | **Total bacteria count/ml** | **Live** | **Dead** | **OD**  **(420nm)** |
| --- | --- | --- | --- | --- | --- |
| **KR40** | 0 | 7,6x10^7^ | 98% | 2% | 0,395 |
|  | 24 h | 1,84x10^8^ | 97% | 3% | 0,400 |
| **N116** | 0 | 3,05x10^7^ | 96% | 4% | 0,451 |
|  | 24 h | 4,55x10^8^ | 97% | 3% | 0,426 |
