# Supplementary figures and images for: Divergent Fates of Hardjo Leptospires: Early Transcriptomic Response of Leptospira interrogans in an Ovine Dialysis Membrane Chamber Model
Source: Transbound Emerg Dis. 2026 Apr 9;2026:2998023. doi: 10.1155/tbed/2998023 (PMC13066512; doi:10.1155/tbed/2998023)

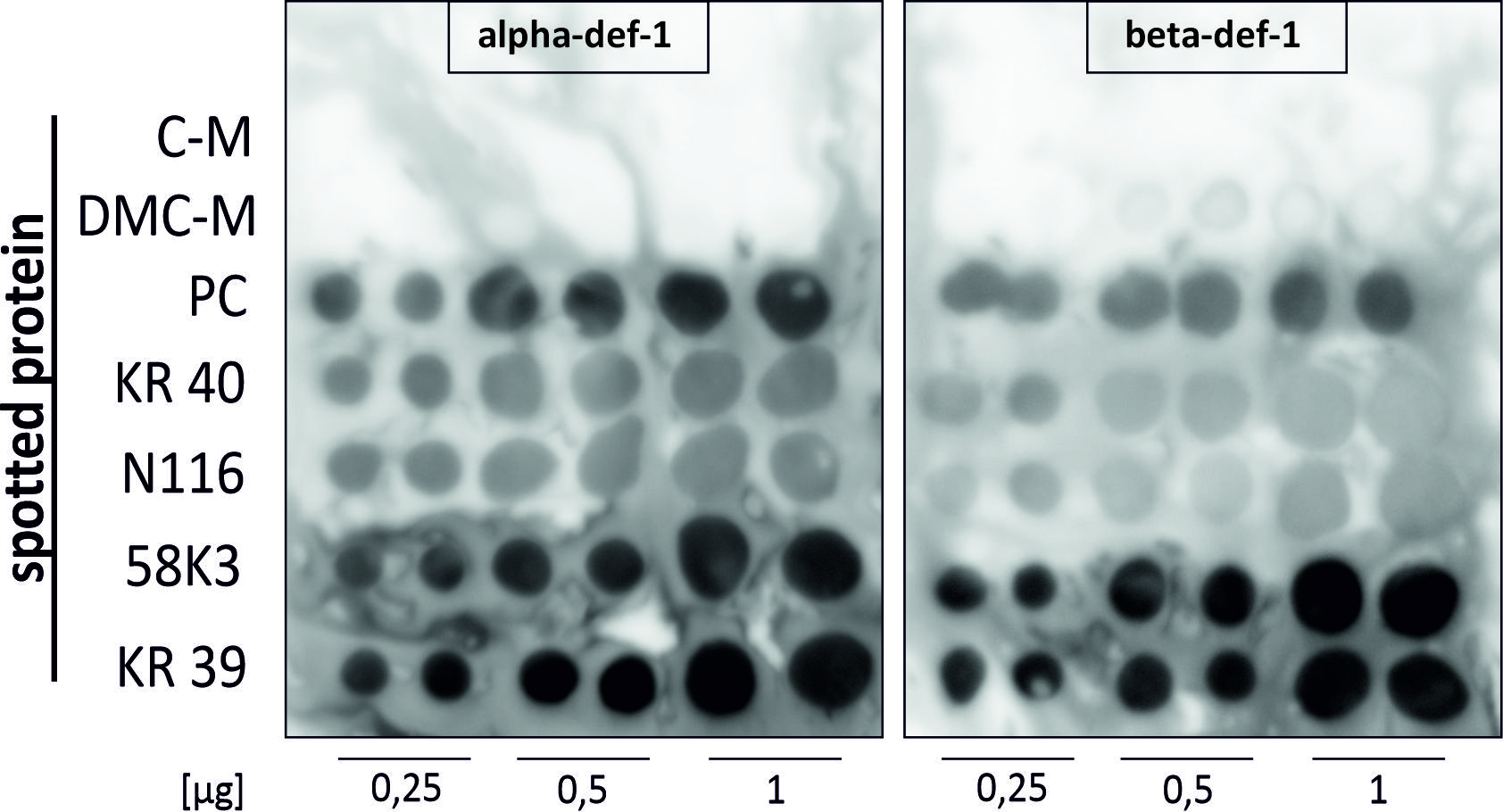

Supplement: Supplementary file 3 — Supporting Information 3 Figure S3: Dot blot detection of host α‐ and β‐defensins in supernatants recovered from DMCs after in vivo incubation in the peritoneal cavity of sheep. Positive control (PC) – purified defensin standard; C‐M – control medium (fresh, non‐incubated culture medium); DMC‐M – incubation medium (medium incubated in DMCs for 24 h without bacteria). [file TBED-2026-2998023-s003.jpg]
