# Supplementary material for: Divergent Fates of Hardjo Leptospires: Early Transcriptomic Response of Leptospira interrogans in an Ovine Dialysis Membrane Chamber Model
Source: Transbound Emerg Dis. 2026 Apr 9;2026:2998023. doi: 10.1155/tbed/2998023 (PMC13066512; doi:10.1155/tbed/2998023)
Supplement: Supplementary file 4 — Supporting Information 4 Table S4: Statistics on sequencing reads and genome alignment in L. interrogans sv. Hardjo. [file TBED-2026-2998023-s004.docx]

| **Sample** | **Group** | **Raw reads (pairs)** | **Filtered read s(pairs)** | **Percentage of reads passing filter** | **Percentage of mapped pairs** | **Number of mapped pairs** |
| --- | --- | --- | --- | --- | --- | --- |
| **KR40** | | | | | | |
| 3 | in vitro | 6040565 | 5864627 | 97,09 | 98,88 | 5798943 |
| 12 | in vitro | 4823263 | 4788179 | 99,27 | 99,27 | 4753213 |
| 16 | in vitro | 6114531 | 5936438 | 97,09 | 98,40 | 5841454 |
| 17 | in vivo 24h | 5393936 | 5236832 | 97,09 | 99,55 | 5213266 |
| 21 | in vivo 24h | 10084622 | 8936527 | 88,62 | 97,57 | 8719319 |
| 25 | in vivo 24h | 12654073 | 12285508 | 97,09 | 99,63 | 12240051 |
| All (sum) | NA | 45110990 | 43048111 | NA | NA | 42566246 |
| All (mean) | NA | 7518498 | 7174685 | 96,04 | 98,88 | 7094374 |
| **N116** | | | | | | |
| 4 | in vitro | 6327233 | 6119180 | 96,71 | 99,57 | 6092868 |
| 10 | in vitro | 9162602 | 8861317 | 96,71 | 99,43 | 8810808 |
| 14 | in vitro | 5466134 | 5286396 | 96,71 | 98,83 | 5224546 |
| 38 | in vivo 24h | 10986732 | 10625466 | 96,71 | 98,57 | 10473522 |
| 22 | in vivo 24h | 13094306 | 12663738 | 96,71 | 99,11 | 12551031 |
| 18 | in vivo 24h | 10384288 | 10042831 | 96,71 | 99,63 | 10005673 |
| All (sum) | NA | 55421295 | 53598928 | NA | NA | 53158448 |
| All (mean) | NA | 9236883 | 8933155 | 96,71 | 99,19 | 8859741 |
