# Supplementary material for: Divergent Fates of Hardjo Leptospires: Early Transcriptomic Response of Leptospira interrogans in an Ovine Dialysis Membrane Chamber Model
Source: Transbound Emerg Dis. 2026 Apr 9;2026:2998023. doi: 10.1155/tbed/2998023 (PMC13066512; doi:10.1155/tbed/2998023)
Supplement: Supplementary file 6 — Supporting Information 6 Table S6: Complete list of differentially expressed genes (DEGs) in L. interrogans sv. organised according to the functional categories discussed in the Results section. ᵃGene identifiers based on NCBI RefSeq annotation of L. interrogans sv. Hardjo strain KR40 (GCF_023158895.1); ᵇProtein product description based on combined RefSeq and UniProt annotations; ᶜlog₂ fold change between in vivo and in vitro conditions; ᵈFalse discovery rate; ᵉGenes without orthologues in L. borgpetersenii sv. Hardjo [file TBED-2026-2998023-s006.docx]

| **ORF ID^a^** | **Gene^a^** | **Product^b^** | **log₂FC^c^** | **FDR^d^** | **Absent in** **LbH^e^** |  |
| --- | --- | --- | --- | --- | --- | --- |
| ***Membrane-associated genes. lipoproteins and putative adhesins*** | | | | | | |
| **Upregulated** | | | | | | |
| *MY479_RS08695* |  | Lipoprotein | 2.45 | 2.88E-16 |  |  |
| *MY479_RS02460* | *ligB* | Lipoprotein adhesin LigB | 3.69 | 5.09E-14 |  |  |
| *MY479_RS00790* | *yidC* | Membrane protein insertase YidC | 1.38 | 4.13E-08 |  |  |
| *MY479_RS00455* |  | Lipoprotein | 1.53 | 1.04E-08 | Yes |  |
| *MY479_RS02465* | *ligA* | Lipoprotein adhesin LigA | 4.07 | 6.7E-08 | Yes |  |
| *MY479_RS12790* |  | Lipoprotein | 1.18 | 6.06E-07 |  |  |
| *MY479_RS03055* |  | Lipoprotein | 1.05 | 1.79E-06 |  |  |
| *MY479_RS01835* | *lolA* | Outer-membrane lipoprotein carrier protein LolA | 1.02 | 1.92E-06 |  |  |
| *MY479_RS05285* |  | Outer membrane protein beta-barrel domain-containing protein | 1.08 | 2.49E-06 |  |  |
| *MY479_RS00785* | *yidD* | Membrane protein insertion efficiency factor YidD | 1.72 | 2.50E-06 |  |  |
| *MY479_RS08840* | *mce* | Mammalian cell entry protein Mce | 1.02 | 3.77E-06 |  |  |
| *MY479_RS16695* | *lenF* | Endostatin-like outer membrane lipoprotein LenF | 1.38 | 8.07E-06 | Yes |  |
| *MY479_RS10850* | *lmtA* | lipid A Kdo2 1-phosphate O-methyltransferase | 1.14 | 1.45E-05 |  |  |
| *MY479_RS06420* |  | Conserved hypothetical protein | 1.45 | 4.62E-05 | Yes |  |
| *MY479_RS13150* |  | LIC10774 family surface protein / DUF1565 domain-containing protein | 1.44 | 4.78E-05 | Yes |  |
| *MY479_RS05265* |  | Outer membrane protein | 0.88 | 1.04E-04 | Yes |  |
| *MY479_RS01900* |  | SBBP repeat beta-propeller lipoprotein. LipL53 family | 1.62 | 1.11E-04 | Yes |  |
| *MY479_RS01875* |  | Lipoprotein Lsa21 | 1.74 | 1.12E-04 | Yes |  |
| *MY479_RS15505* | *lenC* | Endostatin-like outer membrane lipoprotein LenC | 1.08 | 2.13E-04 | Yes |  |
| *MY479_RS17710* |  | LA_1326/LA_4305 family lipoprotein | 1.05 | 2.40E-04 |  |  |
| *MY479_RS15790* |  | SBBP repeat beta-propeller lipoprotein. LipL53 family | 1.68 | 2.65E-04 |  |  |
| *MY479_RS07110* |  | SBBP repeat beta-propeller lipoprotein. LipL53 family | 1.37 | 4.06E-04 |  |  |
| *MY479_RS09690* |  | LIC_11695 family lipoprotein | 1.82 | 4.50E-04 | Yes |  |
| *MY479_RS19380* |  | Lipoprotein | 0.94 | 7.74E-04 |  |  |
| *MY479_RS02455* |  | Lipoprotein | 1.66 | 1.22E-03 |  |  |
| *MY479_RS05500* |  | Lipoprotein | 0.97 | 1.48E-03 | Yes |  |
| *MY479_RS15415* |  | TolC family protein | 0.85 | 1.97E-03 | Yes |  |
| *MY479_RS09440* |  | Lipoprotein | 0.94 | 1.84E-03 |  |  |
| *MY479_RS00060* |  | Lipoprotein | 0.62 | 1.85E-03 | Yes |  |
| *MY479_RS17195* |  | Lipoprotein | 0.60 | 2.12E-03 |  |  |
| *MY479_RS17990* |  | lipoprotein | 0.99 | 2.12E-03 | Yes |  |
| *MY479_RS09995* |  | LA_2272/LA_2273 family lipoprotein | 0.54 | 2.15E-03 | Yes |  |
| *MY479_RS13345* |  | Lipoprotein | 1.01 | 2.16E-03 | Yes |  |
| *MY479_RS04310* |  | Collagenase | 1.00 | 2.19E-03 |  |  |
| *MY479_RS02450* |  | Bor/Iss family lipoprotein | 1.22 | 2.22E-03 |  |  |
| *MY479_RS01890* |  | SBBP repeat beta-propeller lipoprotein. LipL53 family | 1.29 | 2.82E-03 | Yes |  |
| *MY479_RS05050* | *lenB* | endostatin-like outer membrane protein LenB | 0.81 | 3.87E-03 | Yes |  |
| *MY479_RS04695* |  | Lipoprotein | 1.19 | 3.89E-03 | Yes |  |
| *MY479_RS16730* |  | Lipoprotein | 1.01 | 4.37E-03 |  |  |
| *MY479_RS02145* |  | Lipoprotein | 0.80 | 4.82E-03 | Yes |  |
| *MY479_RS02705* |  | Lipoprotein | 0.66 | 5.18E-03 | Yes |  |
| *MY479_RS02610* |  | Lipoprotein (LipL-46 like) | 1.01 | 7.51E-03 | Yes |  |
| *MY479_RS16835* |  | Lipoprotein | 0.79 | 9.08E-03 |  |  |
| *MY479_RS11280* | *spsE* | N-acetylneuraminic (Sialic) acid synthetase | 0.76 | 1.17E-02 |  |  |
| *MY479_RS04875* |  | Lipoprotein | 0.53 | 1.06E-02 | Yes |  |
| *MY479_RS01615* |  | LA_0364 family Cys-rich lipoprotein | 1.25 | 1.21E-02 | Yes |  |
| *MY479_RS07440* |  | FdtA/QdtA family cupin domain-containing protein | 1.27 | 1.45E-02 |  |  |
| *MY479_RS15850* |  | Outer membrane protein | 0.55 | 1.66E-02 | Yes |  |
| *MY479_RS11440* |  | Outer membrane protein | 0.58 | 1.74E-02 |  |  |
| *MY479_RS05495* |  | Lipoprotein | 0.85 | 1.84E-02 | Yes |  |
| *MY479_RS19365* |  | Lipoprotein | 0.64 | 1.87E-02 |  |  |
| *MY479_RS06410* |  | MXAN_6521/LA_1396 family lipoprotein | 1.14 | 1.90E-02 | Yes |  |
| *MY479_RS02645* |  | Lipoprotein | 1.10 | 2.02E-02 | Yes |  |
| *MY479_RS10015* |  | Lipoprotein | 0.49 | 2.14E-02 |  |  |
| *MY479_RS08410* |  | Lipoprotein | 0.91 | 2.15E-02 |  |  |
| *MY479_RS04095* |  | Lipoprotein | 0.89 | 2.63E-02 |  |  |
| *MY479_RS02620* |  | TolC family protein | 1.02 | 2.82E-02 | Yes |  |
| *MY479_RS19480* |  | TIGR04454 family lipoprotein | 0.57 | 2.94E-02 |  |  |
| *MY479_RS10295* |  | Lipoprotein | 0.56 | 4.63E-02 | Yes |  |
| *MY479_RS17245* |  | Lipoprotein | 0.74 | 4.87E-02 | Yes |  |
| *MY479_RS08540* |  | Lipoprotein | 0.47 | 4.95E-02 |  |  |
| *MY479_RS11730* |  | Lipoprotein | 0.43 | 4.98E-02 |  |  |
| **Downregulated** | | | | | | |
| *MY479_RS13930* |  | DUF1566 domain-containing protein | -3.38 | 4.98E-50 |  |  |
| *MY479_RS15970* |  | SbsA Ig-like domain-containing protein | -3.84 | 1.17E-44 | Yes |  |
| *MY479_RS19795* |  | RHS repeat-associated core domain-containing protein | -4..06 | 3.12E-39 | Yes |  |
| *MY479_RS19790* |  | Lipoprotein | -3.18 | 5.09E-14 | Yes |  |
| *MY479_RS15785* | *lipL36* | Lipoprotein LipL36 | -1.60 | 1.56E-07 |  |  |
| *MY479_RS00190* |  | Lipoprotein | -1.50 | 4.73E-06 | Yes |  |
| *MY479_RS15810* |  | Lipoprotein | -1.27 | 1.05E-05 | Yes |  |
| *MY479_RS15770* |  | Lipoprotein | -1.42 | 1.98E-05 |  |  |
| *MY479_RS06440* |  | Lipoprotein | -1.77 | 2.14E-05 | Yes |  |
| *MY479_RS16235* | *lpxA* | acyl-ACP--UDP-N-acetylglucosamine O-acyltransferase | -0.82 | 2.69E-05 |  |  |
| *MY479_RS16220* |  | TIGR04452 family lipoprotein | -1.54 | 4.28E-05 |  |  |
| *MY479_RS10565* | *lptB* | LPS export ABC transporter ATP-binding protein | -0.76 | 1.21E-04 |  |  |
| *MY479_RS10440* | *gspC* | General secretion pathway protein GspC | -1.16 | 1.66E-04 |  |  |
| *MY479_RS00660* |  | Lipoprotein | -1.39 | 2.41E-04 |  |  |
| *MY479_RS19780* |  | Lipoprotein | -1.09 | 2.70E-04 |  |  |
| *MY479_RS12245* |  | Lipoprotein | -0.75 | 3.06E-04 | Yes |  |
| *MY479_RS10405* | *gdpJ* | type II secretion system protein GspJ | -0.87 | 5.07E-04 |  |  |
| *MY479_RS14715* |  | Lipoprotein | -1.07 | 7.16E-04 | Yes |  |
| *MY479_RS15080* |  | Lipoprotein | -0.92 | 1.08E-03 | Yes |  |
| *MY479_RS16690* |  | Lipoprotein | -1.16 | 1.11E-03 |  |  |
| *MY479_RS10385* | *gspN* | type II secretion system protein GspN | -0.59 | 1.22E-03 |  |  |
| *MY479_RS05610* |  | Lipoprotein | -0.96 | 1.42E-03 | Yes |  |
| *MY479_RS10410* |  | type II secretion system protein | -0.92 | 1.55E-03 |  |  |
| *MY479_RS01865* |  | DUF1565 domain-containing protein | -1.70 | 2.20E-03 | Yes |  |
| *MY479_RS01095* |  | Lipoprotein | -0.71 | 2.89E-03 | Yes |  |
| *MY479_RS06600* |  | Lipoprotein | -0.84 | 2.91E-03 |  |  |
| *MY479_RS17530* |  | Lipoprotein | -0.83 | 3.40E-03 |  |  |
| *MY479_RS17775* |  | Lipoprotein | -0.77 | 4.21E-03 |  |  |
| *MY479_RS01610* |  | LA_0364 family Cys-rich lipoprotein | -1.16 | 5.72E-03 | Yes |  |
| *MY479_RS06155* | *lspA* | lipoprotein signal peptidase | -0.74 | 6.06E-03 |  |  |
| *MY479_RS10425* | *gspF* | type II secretion system F family protein | -0.84 | 6.46E-03 |  |  |
| *MY479_RS09370* |  | LIC11755 family lipoprotein | -0.88 | 7.88E-03 |  |  |
| *MY479_RS08920* | *nfeD* | Nodulation efficiency protein NfeD | -1.03 | 8.27E-03 |  |  |
| *MY479_RS10400* | *gspK* | Type II secretion system protein GspK | -0.63 | 9.91E-03 |  |  |
| *MY479_RS14675* |  | Lipoprotein | -0.95 | 1.11E-02 | Yes |  |
| *MY479_RS11510* | *lipL32* | Major surface lipoprotein LipL32 | -1.09 | 1.12E-02 |  |  |
| *MY479_RS14800* |  | TIGR04452 family lipoprotein | -1.69 | 1.15E-02 | Yes |  |
| *MY479_RS02880* |  | Lipoprotein | -0.69 | 1.37E-02 |  |  |
| *MY479_RS12090* |  | Outer membrane protein | -0.98 | 1.44E-02 |  |  |
| *MY479_RS01215* |  | Lipoprotein | -0.49 | 1.46E-02 |  |  |
| *MY479_RS07065* |  | Lipoprotein | -1.23 | 1.98E-02 |  |  |
| *MY479_RS15280* |  | Lipoprotein | -0.78 | 2.03E-02 |  |  |
| *MY479_RS10430* | *gspE* | type II secretion system ATPase GspE | -0.64 | 2.05E-02 |  |  |
| *MY479_RS12915* |  | Lipoprotein | -0.50 | 2.19E-02 | Yes |  |
| *MY479_RS02030* |  | Lipoprotein | -0.63 | 2.98E-02 | Yes |  |
| *MY479_RS14340* |  | Surface antigen OrfC lipoprotein | -0.56 | 3.19E-02 |  |  |
| *MY479_RS00975* |  | Lipoprotein | -1.29 | 4.14E-02 | Yes |  |
| *MY479_RS06605* |  | TolC family protein | -0.48 | 4.63E-02 |  |  |
| *MY479_RS03150* |  | Lipoprotein | -1.94 | 4.80E-02 | Yes |  |
| *MY479_RS02980* | *lpxK* | tetraacyldisaccharide 4'-kinase | -0.72 | 5.00E-02 |  |  |
| ***Iron-related genes*** | | | | | | |
| **Upregulated** | | | | | | |

| *MY479_RS08695* |  | | Lipoprotein | 2.45 | 2.88E-16 |  |
| --- | --- | --- | --- | --- | --- | --- |
| *MY479_RS13360* |  | | Hypothetical protein | 2.81 | 1.61E-14 | Yes |
| *MY479_RS13365* |  | | TonB-dependent receptor | 1.91 | 1.45E-11 |  |
| *MY479_RS14660* |  | | Di-heme oxidoredictase family protein | 1.62 | 2.18E-10 |  |
| *MY479_RS14655* | *lruB* | | Imelysin LruB | 1.92 | 5.39E-10 |  |
| *MY479_RS09695* |  | | TonB-dependent receptor | 1.67 | 5.96E-06 |  |
| *MY479_RS14665* |  | | Imelysin family protein | 1.07 | 2.08E-05 |  |
| *MY479_RS11530* |  | | TonB-dependent receptor | 1.12 | 9.89E-05 | Yes |
| *MY479_RS19660* |  | | TonB-dependent receptor | 0.89 | 1.78E-04 |  |
| *MY479_RS09690* |  | | LIC_11695 family lipoprotein | 1.82 | 4.50E-04 | Yes |
| *MY479_RS17550* |  | | FecR family protein | 0.93 | 6.77E-03 | Yes |
| *MY479_RS19360* |  | | TonB-dependent siderophore receptor | 0.58 | 9.40E-03 |  |
| *MY479_RS15895* |  | | LruC domain-containing protein | 1.11 | 1.35E-02 | Yes |
| *MY479_RS18430* |  | | FecR domain-containing protein | 0.62 | 2.09E-02 |  |
| **Downregulated** | | | | | | |
| *MY479_RS07010* |  | | DUF3015 domain-containing protein | -2.13 | 7.4E-06 | Yes |
| *MY479_RS11895* |  | | Hypothetical protein | -1.29 | 2.89E-05 |  |
| *MY479_RS15530* |  | | DUF2339 domain-containing protein | -1.15 | 7.10E-04 | Yes |
| *MY479_RS11220* | *hemW* | | radical SAM family heme chaperone HemW | -0.60 | 9.27E-03 |  |
| *MY479_RS12095* |  | | Iron dicitrate transport regulator FecR | -1.05 | 2.15E-02 | Yes |
| *MY479_RS07075* |  | | Multicopper oxidase domain-containing protein | -1.18 | 2.34E-02 |  |
| *MY479_RS12550* |  | | FecR domain-containing protein | -1.99 | 2.35E-02 |  |
| *MY479_RS11095* |  | | FecR family protein | -0.61 | 2.93E-02 |  |
| *MY479_RS08965* |  | | FecR domain-containing protein | -0.66 | 3.96E-02 |  |
| ***Stress and redox homeostasis related genes*** | | | | | | |
| **Upregulated** | | | | | | |
| *MY479_RS15680* | |  | DoxX family protein | 2.72 | 6.05E-11 |  |
| *MY479_RS14660* | |  | Di-heme oxidoreductase family protein | 1.62 | 2.18E-10 |  |
| *MY479_RS11795* | |  | putative quinol monooxygenase | 1.31 | 1.26E-06 | Yes |
| *MY479_RS00470* | |  | HEAT repeat domain-containing protein | 1.68 | 1.01E-04 |  |
| *MY479_RS17150* | |  | Ferredoxin | 1.31 | 1.61E-04 |  |
| *MY479_RS17815* | |  | ferredoxin--NADP(+) reductase | 0.78 | 5.94E-03 |  |
| *MY479_RS16445* | |  | DoxX family protein | 0.81 | 8.21E-03 | Yes |
| *MY479_RS18650* | |  | NAD(P)/FAD-dependent oxidoreductase | 0.49 | 9.94E-03 |  |
| *MY479_RS12715* | |  | Cytochrome c peroxidase | 1.03 | 1.87E-02 |  |
| *MY479_RS14860* | |  | Oxidoreductase | 1.48 | 1.40E-02 |  |
| *MY479_RS04715* | |  | Cytochrome c7-like domain-containing protein | 0.41 | 2.62E-02 |  |

| **Downregulated** | | | | | |
| --- | --- | --- | --- | --- | --- |
| *MY479_RS03480* | *mauG1* | Cytochrome c peroxidase | -3.14 | 3.77E-44 |  |
| *MY479_RS03485* | *petE* | Methylamine utilization protein | -1.87 | 2.83E-14 |  |
| *MY479_RS17595* |  | NADH ubiquinone oxidoreductase/hydrogenase 4 subunit G | -2.43 | 3.86E-14 |  |
| *MY479_RS17590* |  | NADH-quinone oxidoreductase subunit D domain-containing protein | -2.30 | 5.09E-14 |  |
| *MY479_RS17580* |  | Formate hydrogenase subunit E | -2.35 | 2.27E-13 |  |
| *MY479_RS03490* |  | Cytochrome c | -1.84 | 2.27E-13 |  |
| *MY479_RS04285* | *tpx* | Thiol peroxidase | -1.37 | 1.11E-11 |  |
| *MY479_RS04390* | *nuoD* | NADH-quinone oxidoreductase subunit D | -1.28 | 1.23E-11 |  |
| *MY479_RS04395* | *nuoC* | NADH-quinone oxidoreductase subunit C | -1.11 | 1.17E-10 |  |
| *MY479_RS17585* |  | NADH ubiquinone complex I subunit/Formate hydrogenase subunit F | -2.12 | 5.46E-09 |  |
| *MY479_RS17575* |  | NADH-quinone oxidoreductase subunit H | -2.24 | 2.45E-08 |  |
| *MY479_RS17570* |  | proton-conducting transporter membrane subunit | -2.25 | 3.56E-08 |  |
| *MY479_RS09085* | *gshA* | Glutamate-cysteine ligase | -1.62 | 8.68E-08 |  |
| *MY479_RS04385* | *nuoE* | NADH-quinone oxidoreductase subunit NuoE | -1.11 | 4.22E-07 |  |
| *MY479_RS13990* |  | thioredoxin domain-containing protein | -1.91 | 4.65E-07 |  |
| *MY479_RS09090* | *gshAB* | bifunctional glutamate—cysteine ligase GshA/glutathione synthetase GshB | -1.22 | 1.07E-06 |  |
| *MY479_RS04370* | *nuoJ* | NADH-quinone oxidoreductase subunit J | -1.05 | 4.12E-06 |  |
| *MY479_RS09075* | *clpA* | ATP-dependent Clp protease ATP-binding subunit ClpA | -1.13 | 7.25E-05 |  |
| *MY479_RS09070* | *clpS* | ATP-dependent Clp protease adapter ClpS | -1.17 | 1.37E-04 |  |
| *MY479_RS09080* | *ggt* | gamma-glutamyltransferase | -1.08 | 1.45E-04 |  |
| *MY479_RS17565* |  | PF13372 domain protein/ alginate export family protein | -2.00 | 2.17E-04 |  |
| *MY479_RS09065* |  | GNAT family N-acetyltransferase | -1.24 | 2.88E-04 |  |
| *MY479_RS04375* | *nuoH* | NADH-quinone oxidoreductase subunit NuoH | -0.95 | 4.16E-04 |  |
| *MY479_RS04355* |  | NADH-quinone oxidoreductase subunit M | -0.76 | 6.53E-04 |  |
| *MY479_RS03685* |  | Cytochrome c | -1.33 | 8.13E-04 |  |
| *MY479_RS18700* |  | thioredoxin family protein | -0.96 | 8.56E-04 |  |
| *MY479_RS16055* |  | MBL fold metallo-hydrolase | -1.45 | 1.28E-03 | Yes |
| *MY479_RS13385* |  | NAD(P)/FAD-dependent oxidoreductase | -1.48 | 1.38E-03 | Yes |
| *MY479_RS18535* | *bcp* | thioredoxin-dependent thiol peroxidase | -1.07 | 2.40E-03 |  |
| *MY479_RS12375* | *perRB* | peroxide-responsive transcriptional repressor PerRB | -1.51 | 2.69E-03 |  |
| *MY479_RS18635* |  | DoxX family protein | -1.09 | 2.78E-03 | Yes |
| *MY479_RS04365* | *nuoK* | NADH-quinone oxidoreductase subunit NuoK | -0.86 | 4.06E-03 |  |
| *MY479_RS04380* | *nuoF* | NADH-quinone oxidoreductase subunit NuoF | -0.92 | 5.24E-03 |  |
| *MY479_RS14265* |  | glutathione S-transferase family protein | -2.33 | 6.43E-03 |  |
| *MY479_RS14210* |  | Thioredoxin | -0.93 | 3.77E-03 |  |
| *MY479_RS11190* | *clpX* | ATP-dependent Clp protease ATP-binding subunit ClpX | -0.43 | 1.03E-02 |  |
| *MY479_RS15855* |  | DoxX family protein | -0.85 | 1.28E-02 |  |
| *MY479_RS18580* |  | Ferredoxin | -0.86 | 2.11E-02 |  |
| *MY479_RS11195* | *clpP* | ATP-dependent Clp endopeptidase proteolytic subunit ClpP | -0.39 | 4.00E-02 |  |
| *MY479_RS08705* |  | c-type cytochrome | -0.58 | 4.09E-02 | Yes |
| ***Signal transduction genes*** | | | | | |
| **Upregulated** | | | | | |
| *MY479_RS18305* |  | histidine kinase N-terminal 7TM domain-containing protein | 0.89 | 6.97E-06 |  |
| *MY479_RS14580* | *rsbU2* | Serine phosphatase RsbU | 0.99 | 1.50E-05 |  |
| *MY479_RS18145* | *mazE* | Transcriptional regulator/antitoxin MazE | 1.25 | 5.96E-04 | Yes |
| *MY479_RS18150* | *mazF* | endoribonuclease MazF | 1.06 | 7.08E-03 | Yes |
| *MY479_RS10545* |  | Histidine kinase | 0.46 | 8.27E-03 |  |
| *MY479_RS05030* | *kdpD* | Histidine kinase Kdpd | 0.48 | 1.96E-02 | Yes |
| *MY479_RS04500* |  | type II toxin-antitoxin system PemK/MazF family toxin | 0.81 | 4.27E-02 | Yes |
| **Downregulated** | | | | | |
| *MY479_RS18470* |  | Cyclic diguanylate phosphodiesterase | -3.10 | 2.18E-59 | Yes |
| *MY479_RS19535* |  | STAS domain-containing protein | -5.84 | 5.16E-34 |  |
| *MY479_RS18460* |  | STAS domain-containing protein | -2.56 | 8.99E-29 | Yes |
| *MY479_RS18455* |  | Hypothetical protein | -2.84 | 2.71E-25 |  |
| *MY479_RS18450* |  | Tetratricopeptide repeat protein | -2.61 | 6.73E-19 |  |
| *MY479_RS18445* | *rsbU* | Serine phosphatase RsbU | -1.73 | 8.13E-11 |  |
| *MY479_RS12210* |  | Cyclic diguanylate phosphodiesterase | -2.08 | 1.56E-08 |  |
| *MY479_RS05570* |  | Cyclic diguanylate phosphodiesterase | -1.09 | 2.66E-08 |  |
| *MY479_RS12505* |  | Diguanylate cyclase | -1.89 | 4.21E-08 | Yes |
| *MY479_RS07660* |  | PAS domain S-box protein | -1.30 | 8.74E-08 | Yes |
| *MY479_RS13855* |  | Adenylate/guanylate cyclase | -1.54 | 2.30E-07 | Yes |
| *MY479_RS19530* |  | Cyclic diguanylate phosphodiesterase | -1.34 | 6.14E-07 |  |
| *MY479_RS11110* |  | Histidine kinase | -1.09 | 1.06E-06 |  |
| *MY479_RS18465* |  | STAS domain-containing protein | -5.34 | 1.07E-06 | Yes |
| *MY479_RS12220* |  | Response regulator | -2.30 | 2.79E-06 |  |
| *MY479_RS12225* |  | Histidine kinase | -2.03 | 2.79E-06 | Yes |
| *MY479_RS12215* |  | Histidine kinase | -2.13 | 3.96E-06 |  |
| *MY479_RS17210* |  | Histidine kinase | -1.10 | 4.60E-06 |  |
| *MY479_RS12515* |  | Diguanylate cyclase | -1.51 | 6.79E-06 |  |
| *MY479_RS13605* |  | Histidine kinase | -0.92 | 6.97E-06 | Yes |
| *MY479_RS12680* | *cyaA* | Adenylate/guanylate cyclase | -1.31 | 9.41E-06 | Yes |
| MY479_RS12235 | *cyaA5* | Adenylate/guanylate cyclase | -0.92 | 1.93E-05 | Yes |
| *MY479_RS19525* |  | Diguanylate cyclase | -1.23 | 2.74E-05 |  |
| *MY479_RS06965* |  | Histidine kinase | -1.02 | 7.39E-05 |  |
| *MY479_RS14270* |  | Histidine kinase | -1.62 | 1.12E-04 |  |
| *MY479_RS00515* |  | Histidine kinase | -0.69 | 1.43E-04 |  |
| *MY479_RS07655* |  | response regulator | -0.97 | 2.61E-04 |  |
| *MY479_RS10645* |  | Histidine kinase | -1.99 | 3.53E-04 |  |
| *MY479_RS04180* |  | Anti-sigma factor antagonist | -0.78 | 4.75E-04 |  |
| *MY479_RS04480* |  | Response regulator with HD-GYP domain | -0.90 | 4.93E-04 |  |
| *MY479_RS16455* |  | Adenylate/guanylate cyclase | -1.00 | 5.53E-04 | Yes |
| *MY479_RS12520* |  | Diguanylate cyclase | -1.12 | 7.57E-04 | Yes |
| *MY479_RS07740* |  | Antisigma factor antagonist-related protein | -0.80 | 1.06E-03 |  |
| *MY479_RS12485* |  | Two-component response regulator | -0.76 | 1.23E-03 | Yes |
| *MY479_RS01365* |  | Cyclic nucleotide-binding domain-containing protein | -0.53 | 1.25E-03 |  |
| *MY479_RS13600* |  | Two-component response regulator receiver protein | -0.72 | 1.29E-03 |  |
| *MY479_RS06160* | *crp* | cAMP-binding protein | -0.92 | 2.13E-03 |  |
| *MY479_RS11055* | *pleD* | Diguanylate cyclase | -0.63 | 2.13E-03 |  |
| *MY479_RS12510* |  | Diguanylate cyclase | -1.04 | 2.15E-03 | Yes |
| *MY479_RS17145* |  | Histidine kinase | -0.87 | 2.52E-03 |  |
| *MY479_RS11115* |  | Histidine kinase | -0.92 | 3.29E-03 |  |
| *MY479_RS06780* |  | Diguanylate cyclase | -0.98 | 4.27E-03 |  |
| *MY479_RS07055* |  | Histidine kinase | -1.08 | 5.22E-03 | Yes |
| *MY479_RS12125* |  | Histidine kinase | -0.59 | 5.39E-03 |  |
| *MY479_RS07795* |  | Histidine kinase | -0.68 | 6.83E-03 |  |
| *MY479_RS05485* |  | Serine/threonine kinase | -0.57 | 9.34E-03 | Yes |
| *MY479_RS00405* |  | STAS domain-containing protein | -1.22 | 1.06E-02 |  |
| *MY479_RS07790* |  | Histidine kinase | -0.71 | 1.21E-02 |  |
| *MY479_RS16065* |  | Cyclic diguanylate phosphodiesterase | -1.16 | 1.45E-02 |  |
| *MY479_RS06525* | *ptc1* | Serine/threonine-protein phosphatase | -0.95 | 1.55E-02 |  |
| *MY479_RS02835* |  | Cyclic diguanylate phosphodiesterase | -0.83 | 1.65E-02 |  |
| *MY479_RS10660* |  | STAS domain-containing protein | -1.89 | 2.55E-02 |  |
| *MY479_RS12700* |  | STAS domain-containing protein | -0.92 | 2.65E-02 |  |
| *MY479_RS10715* |  | Serine/threonine phosphatase containing GAF domains | -0.47 | 2.87E-02 |  |
| *MY479_RS13210* |  | STAS domain-containing protein | -0.67 | 2.94E-02 |  |
| *MY479_RS11080* |  | Histidine kinase | -1.35 | 3.26E-02 | Yes |
| *MY479_RS10710* |  | Anti-sigma factor antagonist | -0.51 | 4.28E-02 |  |
| *MY479_RS17140* |  | Response regulator | -0.56 | 4.67E-02 |  |
| *MY479_RS13435* |  | Serine/threonine phosphatase containing GAF domains | -0.58 | 4.73E-02 |  |
| ***Chemotaxis and motility genes*** | | | | | |
| **Upregulated** | | | | | |

| *MY479_RS11245* |  | Methyl-accepting chemotaxis protein | 2.44 | 4.39E-16 |  |  |
| --- | --- | --- | --- | --- | --- | --- |
| *MY479_RS00225* |  | Methyl-accepting chemotaxis protein | 1.04 | 3.06E-04 |  |  |
| *MY479_RS15195* |  | Flagellin FlaB | 0.87 | 6.88E-03 |  |  |
| *MY479_RS15755* |  | Flagellar motor protein MotB | 0.64 | 9.40E-03 |  |  |
| *MY479_RS01260* |  | Flagellar motor switch protein FliG C-terminal domain-containing protein | 1.19 | 1.45E-02 |  |  |
| *MY479_RS14205* |  | FcpA-related putative periplasmic flagellar protein | 0.52 | 3.79E-02 |  |  |
| *MY479_RS09710* |  | Methyl-accepting chemotaxis protein | 0.45 | 3.45E-02 | Yes |  |
| **Downregulated** | | | | | | |
| *MY479_RS03495* |  | Methyl-accepting chemotaxis protein | -1.73 | 1.20E-19 |  |  |
| *MY479_RS17480* |  | Methyl-accepting chemotaxis protein | -1.34 | 5.29E-08 |  |  |
| *MY479_RS07780* | *cheR* | Protein-glutamate O-methyltransferase CheR | -1.89 | 5.48E-06 |  |  |
| *MY479_RS03650* |  | Flagellar FlbD family protein | -1.00 | 6.85E-05 |  |  |
| *MY479_RS16360* |  | Chemotaxis protein | -1.44 | 1.58E-03 |  |  |
| *MY479_RS00125* | *fliG* | flagellar motor switch protein FliG | -0.68 | 3.32E-03 |  |  |
| *MY479_RS12305* |  | Flagellar Assembly Protein A | -1.00 | 7.08E-03 |  |  |
| *MY479_RS10675* | *cheW* | chemotaxis protein CheW | -2.30 | 1.21E-02 |  |  |
| *MY479_RS04470* | *flgD* | flagellar hook capping FlgD | -0.72 | 1.35E-02 |  |  |
| *MY479_RS14595* |  | flagellar filament capping protein FliD | -0.70 | 1.43E-02 |  |  |
| *MY479_RS04475* | *flgE* | flagellar hook protein FlgE | -0.77 | 1.51E-02 |  |  |
| *MY479_RS10685* | *cheB* | chemotaxis protein CheB | -1.18 | 1.82E-02 |  |  |
| *MY479_RS17735* | *flgN* | flagellar protein FlgN | -0.60 | 2.15E-02 |  |  |
| *MY479_RS10665* | *cheA* | chemotaxis protein CheA | -1.81 | 2.24E-02 | Yes |  |
| *MY479_RS11620* | *flgA* | flagellar basal body P-ring formation chaperone FlgA | -0.72 | 2.26E-02 |  |  |
| *MY479_RS10680* | *cheD* | chemotaxis protein CheD | -1.93 | 2.27E-02 |  |  |
| *MY479_RS03655* | *motA* | motility protein A | -0.56 | 2.61E-02 |  |  |
| *MY479_RS11605* | *flgJ* | rod-binding protein FlgJ | -0.61 | 2.76E-02 |  |  |
| *MY479_RS10655* | *cheY* | Chemotaxis protein CheY | -1.41 | 3.03E-02 |  |  |
| *MY479_RS01530* | *fliE* | flagellar hook-basal body complex protein FliE | -0.58 | 2.98E-02 |  |  |
| *MY479_RS11610* | *flgI* | flagellar basal body P-ring protein FlgI | -0.72 | 4.38E-02 |  |  |
| *MY479_RS11420* | *fliO* | flagellar biosynthetic protein FliO | -0.49 | 4.62E-02 |  |  |

| ***Energy and metabolism genes*** | | | | | |
| --- | --- | --- | --- | --- | --- |
| **Upregulated** | | | | | |
| *MY479_RS15425* | *sul1* | Carbonic anhydrase | 3.18 | 1.72E-13 |  |
| *MY479_RS08145* |  | PaaI family thioesterase | 1.18 | 1.39E-11 |  |
| *MY479_RS07325* |  | DapH/DapD/GlmU-related protein | 1.10 | 9.78E-08 |  |
| *MY479_RS19055* |  | Glycosyltransferase RgtA/B/C/D-like domain-containing protein | 1.41 | 1.91E-07 |  |
| *MY479_RS12025* | *atpH* | ATP synthase F1 subunit delta | 1.13 | 2.39E-06 |  |
| *MY479_RS07255* |  | DegT/DnrJ/EryC1/StrS aminotransferase family protein | 1.22 | 2.79E-06 |  |
| *MY479_RS12035* | *aptE* | ATP synthase F0 subunit C | 1.23 | 5.91E-06 |  |
| *MY479_RS12030* | *atpF* | F0F1 ATP synthase subunit B | 1.10 | 9.11E-06 |  |
| *MY479_RS04540* |  | Glycosyltransferase family 4 protein | 1.14 | 1.15E-04 |  |
| *MY479_RS09680* |  | Polysaccharide deacetylase family protein | 0.84 | 1.21E-04 |  |
| *MY479_RS07375* | *wcaG3* | Surface carbohydrate biosynthesis protein | 0.92 | 1.38E-04 |  |
| *MY479_RS18615* |  | polysaccharide deacetylase family protein | 0.79 | 2.59E-04 |  |
| *MY479_RS12020* | *atpA* | F0F1 ATP synthase subunit alpha | 0.96 | 3.87E-04 |  |
| *MY479_RS00605* | *thiD* | bifunctional hydroxymethylpyrimidine kinase/phosphomethylpyrimidine kinase | 0.96 | 5.80E-04 |  |
| *MY479_RS01635* | *rpiB* | ribose 5-phosphate isomerase B | 1.06 | 7.50E-04 |  |
| *MY479_RS19205* | *alr* | Alanine racemase | 1.01 | 9.40E-04 |  |
| *MY479_RS06065* | *glnA* | **Glutamine synthetase** | 1.12 | 1.12E-03 |  |
| *MY479_RS10975* |  | Triacylglycerol lipase | 1.87 | 1.01E-03 |  |
| *MY479_RS09620* | *fbp* | class 1 fructose-bisphosphatase | 1.41 | 1.03E-03 |  |
| *MY479_RS07280* |  | FkbM family methyltransferase | 1.13 | 1.12E-03 |  |
| *MY479_RS16570* | *ppa* | inorganic diphosphatase | 0.82 | 1.16E-03 |  |
| *MY479_RS11235* | *ilvB* | biosynthetic-type acetolactate synthase large subunit | 0.93 | 1.44E-03 |  |
| *MY479_RS16575* |  | Glycerol-3-phosphate antiporter | 1.20 | 1.53E-03 |  |
| *MY479_RS12010* | *atpD* | F0F1 ATP synthase subunit beta | 0.90 | 1.88E-03 |  |
| *MY479_RS12040* | *atpB* | F0F1 ATP synthase subunit A | 1.05 | 2.28E-03 |  |
| *MY479_RS01895* |  | Glycosyltransferase RgtA/B/C/D-like domain-containing protein | 1.23 | 2.45E-03 | Yes |
| *MY479_RS07125* | *wcaG2* | GDP-L-fucose synthase | 0.94 | 2.94E-03 |  |
| *MY479_RS05985* |  | Aminotransferase. class I/II | 1.02 | 3.10E-03 |  |
| *MY479_RS07205* |  | D-alanine--D-alanine ligase | 0.83 | 3.42E-03 |  |
| *MY479_RS10590* | *rfaE2* | D-glycero-beta-D-manno-heptose 1-phosphate adenylyltransferase | 0.92 | 4.10E-03 |  |
| *MY479_RS12575* |  | alpha-glucosidase | 0.58 | 4.38E-03 | Yes |
| *MY479_RS07295* | *kdsB* | 3-deoxy-manno-octulosonate cytidylyltransferase | 0.77 | 4.56E-03 |  |
| *MY479_RS02255* | *glmM* | phosphoglucosamine mutase | 0.73 | 5.22E-03 |  |
| *MY479_RS02160* | *ispE* | 4-(cytidine 5'-diphospho)-2-C-methyl-D-erythritol kinase | 0.97 | 5.45E-03 |  |
| *MY479_RS11025* | *hisD* | histidinol dehydrogenase | 0.68 | 6.27E-03 |  |
| *MY479_RS14990* | *lysC* | aspartate kinase | 0.96 | 7.11E-03 |  |
| *MY479_RS06535* | *sch* | Beta-ketoacyl synthase | 1.03 | 8.04E-03 |  |
| *MY479_RS17275* | *cobS* | adenosylcobinamide-GDP ribazoletransferase | 0.76 | 9.58E-03 |  |
| *MY479_RS12015* | *atpG* | ATP synthase F1 subunit gamma | 0.65 | 1.08E-02 |  |
| *MY479_RS07250* |  | WbqC family protein | 0.78 | 1.06E-02 |  |
| *MY479_RS05875* | *hisG* | ATP phosphoribosyltransferase | 0.57 | 1.17E-02 |  |
| *MY479_RS10055* | *fsa* | fructose-6-phosphate aldolase | 0.51 | 1.22E-02 |  |
| *MY479_RS06505* | *fabH* | 3-oxoacyl-[acyl-carrier-protein] synthase | 1.01 | 1.26E-02 |  |
| *MY479_RS12005* | *atpC* | ATP synthase F1 subunit epsilon | 0.62 | 1.36E-02 |  |
| *MY479_RS10640* | *ispH* | 4-hydroxy-3-methylbut-2-enyl diphosphate reductase | 0.54 | 1.42E-02 |  |
| *MY479_RS07440* |  | FdtA/QdtA family cupin domain-containing protein | 1.27 | 1.45E-02 |  |
| *MY479_RS11475* |  | MaoC family dehydratase | 0.54 | 1.50E-02 |  |
| *MY479_RS07290* | *hpcH* | 6-phosphogluconolactonase | 0.72 | 1.64E-02 |  |
| *MY479_RS02175* | *prs* | ribose-phosphate pyrophosphokinase | 0.83 | 2.49E-02 |  |
| *MY479_RS18400* | *cbiB* | adenosylcobinamide-phosphate synthase CbiB | 0.72 | 2.72E-02 |  |
| *MY479_RS07330* |  | Inositol monophosphatase | 0.45 | 2.94E-02 |  |
| *MY479_RS18370* | *cobM* | precorrin-4 C(11)-methyltransferase | 0.78 | 3.36E-02 |  |
| *MY479_RS12255* | *citE* | CoA ester lyase | 0.53 | 3.47E-02 |  |
| *MY479_RS18360* | *cbiG* | cobalt-precorrin 5A hydrolase | 0.87 | 3.56E-02 |  |
| *MY479_RS07180* |  | DegT/DnrJ/EryC1/StrS aminotransferase family protein | 0.72 | 3.99E-02 |  |
| *MY479_RS09035* | *leuC* | 3-isopropylmalate dehydratase large subunit | 0.53 | 4.46E-02 |  |
| **Downregulated** | | | | | |
| *MY479_RS15970* |  | Nucleoside-diphosphate sugar epimerase | -3.84 | 1.17E-44 | Yes |
| *MY479_RS01855* | *ivd* | Isovaleryl-CoA dehydrogenase | -1.41 | 3.09E-08 |  |
| *MY479_RS00195* |  | **GMC oxidoreductase** | -1.40 | 9.78E-08 | Yes |
| *MY479_RS05405* | *ribE* | riboflavin synthase | -1.37 | 3.65E-07 |  |
| *MY479_RS09950* | *glpA2* | Glycerol-3-phosphate dehydrogenase | -1.42 | 2.57E-07 | Yes |
| *MY479_RS09390* |  | Glycosyltransferase | -1.66 | 3.65E-07 |  |
| *MY479_RS11435* | *asd* | archaetidylserine decarboxylase | -0.85 | 4.45E-06 |  |
| *MY479_RS04530* |  | VOC family protein | -1.30 | 4.79E-06 | Yes |
| *MY479_RS04635* | *trpA* | tryptophan synthase subunit alpha | -1.05 | 6.02E-06 |  |
| *MY479_RS16415* |  | alpha/beta fold hydrolase | -1.63 | 1.40E-05 | Yes |
| *MY479_RS05255* | *gspA* | 1-acyl-sn-glycerol-3-phosphate acyltransferase | -1.05 | 1.79E-05 |  |
| *MY479_RS03035* | *caiA1* | Acyl-CoA dehydrogenase | -1.01 | 2.89E-05 |  |
| *MY479_RS17495* | *Acs* | acetate--CoA ligase | -1.13 | 4.82E-05 |  |
| *MY479_RS00380* | *dapF* | diaminopimelate epimerase | -1.13 | 8.20E-05 |  |
| *MY479_RS04185* | *trpC* | indole-3-glycerol-phosphate synthase | -0.75 | 9.93E-05 |  |
| *MY479_RS05040* |  | alpha/beta hydrolase | -1.85 | 1.01E-04 |  |
| *MY479_RS16420* |  | **Cholesterol oxidase** | -1.31 | 1.04E-04 | Yes |
| *MY479_RS00285* | *cysE* | serine O-acetyltransferase | -1.02 | 1.63E-04 |  |
| *MY479_RS03360* | *thiL* | thiamine-phosphate kinase | -0.84 | 5.24E-04 |  |
| *MY479_RS10970* |  | alpha/beta hydrolase | -1.32 | 4.63E-04 | Yes |
| *MY479_RS05375* | *trpD* | anthranilate phosphoribosyltransferase | -0.73 | 7.25E-04 |  |
| *MY479_RS01030* | *suhB2* | inositol monophosphatase family protein | -0.84 | 7.70E-04 |  |
| *MY479_RS14160* |  | enoyl-CoA hydratase/isomerase family protein | -0.63 | 9.98E-04 |  |
| *MY479_RS05625* | *crt* | 3-hydroxybutyryl-CoA dehydratase | -0.74 | 1.09E-03 |  |
| *MY479_RS04630* | *trpB* | tryptophan synthase subunit beta | -0.86 | 1.13E-03 |  |
| *MY479_RS15545* | *hisI* | phosphoribosyl-AMP cyclohydrolase | -1.41 | 1.21E-03 |  |
| *MY479_RS05410* | *ribB* | bifunctional 3.4-dihydroxy-2-butanone-4-phosphate synthase/GTP cyclohydrolase II | -0.86 | 1.35E-03 |  |
| *MY479_RS14725* | *fabG* | Short chain dehydrogenase | -0.96 | 1.44E-03 |  |
| *MY479_RS03305* |  | alpha/beta hydrolase | -1.31 | 1.71E-03 |  |
| *MY479_RS05400* | *ribD* | bifunctional diaminohydroxyphosphoribosylaminopyrimidine deaminase/5-amino-6-(5-phosphoribosylamino)uracil reductase RibD | -1.00 | 1.89E-03 |  |
| *MY479_RS15395* |  | Lactonizing lipase | -3.26 | 1.92E-03 | Yes |
| *MY479_RS17625* |  | fructosamine kinase | -0.79 | 2.26E-03 |  |
| *MY479_RS19000* | *metF* | methylenetetrahydrofolate reductase [NAD(P)H] | -0.74 | 2.69E-03 |  |
| *MY479_RS02065* | *ribH* | 6.7-dimethyl-8-ribityllumazine synthase | -0.56 | 3.46E-03 |  |
| *MY479_RS16460* |  | GMC oxidoreductase | -1.34 | 4.09E-03 |  |
| *MY479_RS05370* | *pgsA* | CDP-diacylglycerol--glycerol-3-phosphate 3-phosphatidyltransferase | -0.65 | 4.79E-03 |  |
| *MY479_RS12370* | *lpdA* | dihydrolipoyl dehydrogenase (E3) | -1.15 | 5.10E-03 |  |
| *MY479_RS13390* | *adhP3* | Zn-dependent alcohol dehydrogenase | -0.71 | 5.78E-03 |  |
| *MY479_RS08355* | *eno* | phosphopyruvate hydratase | -0.61 | 6.54E-03 |  |
| *MY479_RS18630* | *faa1* | long-chain fatty acid--CoA ligase | -0.69 | 8.35E-03 | Yes |
| *MY479_RS16190* | *gspA2* | NAD(P)H-dependent glycerol-3-phosphate dehydrogenase | -0.55 | 1.30E-02 |  |
| *MY479_RS15205* | *aroE* | shikimate dehydrogenase | -0.65 | 1.05E-02 |  |
| *MY479_RS14200* | *msgA* | methylglyoxal synthase | -0.62 | 1.08E-02 |  |
| *MY479_RS07775* |  | 1-acyl-sn-glycerol-3-phosphate acyltransferase | -0.67 | 1.31E-02 |  |
| *MY479_RS01435* | *adhP2* | medium chain dehydrogenase/reductase family protein | -0.88 | 1.35E-02 | Yes |
| *MY479_RS08755* |  | alpha/beta fold hydrolase | -0.75 | 1.40E-02 |  |
| *MY479_RS01950* |  | Acyl-CoA dehydrogenase | -0.72 | 1.46E-02 |  |
| *MY479_RS02440* |  | VOC family protein | -1.08 | 1.53E-02 | Yes |
| *MY479_RS08630* | *acoB* | pyruvate dehydrogenase complex E1 component subunit beta | -1.22 | 1.57E-02 |  |
| *MY479_RS04415* | *maoC2* | MaoC family dehydratase | -0.54 | 1.90E-02 |  |
| *MY479_RS18505* | *aroK* | shikimate kinase | -0.68 | 1.99E-02 |  |
| *MY479_RS11825* |  | acetyl/propionyl/methylcrotonyl-CoA carboxylase subunit alpha | -0.68 | 2.05E-02 |  |
| *MY479_RS06020* |  | glycerol-3-phosphate 1-O-acyltransferase PlsY | -0.65 | 2.21E-02 |  |
| *MY479_RS11445* |  | sterol desaturase family protein | -0.75 | 2.31E-02 | Yes |
| *MY479_RS00385* | *plsC* | 1-acyl-sn-glycerol-3-phosphate acyltransferase | -0.44 | 2.33E-02 |  |
| *MY479_RS08635* | *pdhA* | pyruvate dehydrogenase (acetyl-transferring) E1 component subunit alpha | -0.96 | 2.52E-02 |  |
| *MY479_RS15990* | *galK* | galactokinase | -0.61 | 2.52E-02 |  |
| *MY479_RS01955* | *gpmI* | 2.3-bisphosphoglycerate-independent phosphoglycerate mutase | -0.61 | 2.68E-02 |  |
| *MY479_RS05555* | *hisC* | histidinol-phosphate transaminase | -0.57 | 2.70E-02 |  |
| *MY479_RS14090* |  | GNAT family N-acetyltransferase | -1.09 | 2.77E-02 |  |
| *MY479_RS09015* | *gmhA* | D-sedoheptulose 7-phosphate isomerase | -0.40 | 3.24E-02 |  |
| *MY479_RS16145* | *caiA8* | acyl-CoA dehydrogenase | -0.74 | 3.47E-02 |  |
| *MY479_RS15135* | *manA* | Mannose-6-phosphate isomerase | -0.54 | 3.66E-02 |  |
